# Development of a local controlled release system for therapeutic proteins in the treatment of skeletal muscle injuries and diseases

**DOI:** 10.1038/s41419-024-06645-2

**Published:** 2024-07-02

**Authors:** Rachel Lev, Orit Bar-Am, Galit Saar, Ombretta Guardiola, Gabriella Minchiotti, Eli Peled, Dror Seliktar

**Affiliations:** 1 Faculty of Biomedical Engineering, Technion - Israel Institute of Technology, Haifa, Israel; 2 Faculty of Medicine, Technion - Israel Institute of Technology, Haifa, Israel; 3 Stem Cell Fate Laboratory, Institute of Genetics and Biophysics “A. Buzzati Traverso”, CNR, Naples, Italy; 4 Rambam Health Care Campus, Haifa, Israel

**Keywords:** Translational research, Muscle stem cells

## Abstract

The present study aims to develop and characterize a controlled-release delivery system for protein therapeutics in skeletal muscle regeneration following an acute injury. The therapeutic protein, a membrane-GPI anchored protein called Cripto, was immobilized in an injectable hydrogel delivery vehicle for local administration and sustained release. The hydrogel was made of poly(ethylene glycol)-fibrinogen (PEG-Fibrinogen, PF), in the form of injectable microspheres. The PF microspheres exhibited a spherical morphology with an average diameter of approximately 100 micrometers, and the Cripto protein was uniformly entrapped within them. The release rate of Cripto from the PF microspheres was controlled by tuning the crosslinking density of the hydrogel, which was varied by changing the concentration of poly(ethylene glycol) diacrylate (PEG-DA) crosslinker. In vitro experiments confirmed a sustained-release profile of Cripto from the PF microspheres for up to 27 days. The released Cripto was biologically active and promoted the in vitro proliferation of mouse myoblasts. The therapeutic effect of PF-mediated delivery of Cripto in vivo was tested in a cardiotoxin (CTX)-induced muscle injury model in mice. The Cripto caused an increase in the in vivo expression of the myogenic markers Pax7, the differentiation makers eMHC and Desmin, higher numbers of centro-nucleated myofibers and greater areas of regenerated muscle tissue. Collectively, these results establish the PF microspheres as a potential delivery system for the localized, sustained release of therapeutic proteins toward the accelerated repair of damaged muscle tissue following acute injuries.

## Introduction

Skeletal muscle injuries and diseases are associated with a wide range of clinical conditions, including diminished motor capacity and physical disability [1, 2]. Effective regeneration of skeletal muscle following minor injury occurs via activation, proliferation, and differentiation of satellite cells, leading to new myofiber formation [3, 4]. However, the regenerative capacity of skeletal muscle can be severely compromised by the onset of chronic muscle degeneration associated with inherited genetic diseases such as muscular dystrophies (MDs), or by severe acute muscle degeneration after laceration injuries [5, 6]. Current conservative treatments for muscle injuries are intended to relieve the pain with pharmacological treatment combined with physiotherapy, whereas more severe cases might involve invasive surgery [7, 8]. Similarly, there are no effective cures available for MDs [4]; However, medications and supportive therapy, such as physiotherapy, steroid medication, and orthopedic surgery, can help manage symptoms and slow the course of the disease [9, 10]. Several innovative approaches to repair muscle injuries are currently under clinical investigation, including gene therapy, pharmacological therapy, and cell therapy [2, 11, 12]. Though promising, these treatments remain several years away from application in human patients. In the absence of any available treatment, there is still an unmet medical need for intermediate therapies that mitigate the pathology associated with acute and chronic muscle damage.

Muscle restoration through pharmacological therapy is one of the holy grails in the treatment of muscle diseases. In this context, one particularly promising approach to drug therapy is the use of exogenous proteins that are essential in the regeneration of skeletal muscles after acute injury and in MDs. Recombinant protein drugs, which are fueling a renewed growth in the industry [13], may hold the most potential for finding a cure to certain muscle diseases. Therapeutic proteins are considered appropriate in terms of safety, minimal toxicity, clinical feasibility, and manufacturing. Proteins that target signaling pathways associated with muscle regeneration can be developed using advanced techniques in biotechnology. A number of protein drugs have been explored as a potential therapeutic technology for the treatment for skeletal muscle injuries and diseases [14]. Previous studies have demonstrated the benefits of therapeutic proteins such as laminin and recombinant MG53 on skeletal muscle regeneration in animal models [15]. Other recombinant proteins, including galectin-1, utrophin and SDF-1α combined with IGF-1, have been found to be effective in muscle tissue repair by exploiting different actions in the regeneration process, including stem cell recruitment, macrophage activation, and more [16–19].

Therapeutic proteins that target the transforming growth factor-β (TGF-β) signaling pathways may be useful in activating skeletal muscle satellite cells towards muscle regeneration [20–22]. TGF-β signaling, which is linked to normal muscle regeneration processes, can impair regeneration when chronically elevated levels of this factor are present in the muscle niche [23]. Myostatin, a member of the TGF-β superfamily, has become a widely investigated target for growth inhibition in muscle disease because it negatively controls regeneration [24, 25]. Recent evidence demonstrates that illuminating the myostatin/ActRIIB signaling pathway lessens the pathology and improves the function of dystrophic muscle in DMD animal models [26]. Among recently reported myostatin inhibitors, one of particular interest for its potential therapeutic value is the Cripto protein. Cripto is a membrane-GPI anchored protein, which belongs to the family of the epidermal growth factor-Cripto-1/FRL-1/Cryptic (EGF–CFC) proteins [27]. Cripto has been demonstrated to play an important regulatory role in embryonic development and stem cell differentiation [28–30]. It functions as a co-receptor of Nodal and several other TGF-β family ligands [31–33]. Previous findings have indicated that Cripto, which is expressed in myoblast cells of regenerating muscles but not in normal muscle fibers, influences myostatin signaling in myoblasts [34]. Guardiola et al. showed that Cripto modulates myogenic cell determination and promotes proliferation by antagonizing myostatin [24]. Specifically, they demonstrated that a recombinant soluble form of Cripto antagonizes the antiproliferative effect of myostatin and promotes the myogenic commitment of satellite cells on isolated myofibers [24]. Furthermore, Prezioso et al. has also demonstrated that over-expression of Cripto in the satellite cell compartment promotes myogenic commitment and differentiation and enhances early muscle regeneration [35]. Angrisano et al. demonstrated that muscle injury was associated with increased Cripto gene expression and decreased myomiR-1 (a muscle-specific microRNA precursor), suggesting that Cripto could be used as a new pharmacological target for muscle repair [36]. Iavarone et al. demonstrated that conditional deletion of Cripto in the myeloid lineage (Cripto(My-LOF)) macrophages perturbs their plasticity in injured and diseased skeletal muscles, restricting Endothelial-to-Mesenchymal Transition (EndMT) and reducing muscle regenerative potential [37]. Guardiola et al. showed that Cripto micro-heterogeneity affects the balance of proliferation, self-renewal, and myogenic commitment in mouse-activated satellite cells, resulting in increased self-renewal in vivo [38]. Based on these results, we seek to deliver exogenous Cripto to muscle injuries as a regulator of muscle regeneration by promoting myogenic progression of satellite cells.

One major obstacle in the pharmaceutical use of Cripto is its limited in vivo stability. Cripto injected in vivo in an aqueous suspension has a short half-life due to the immune response and enzymatic degradation associated with the environment of the muscle injury [39]. However, there is now a growing use of protein encapsulation in semi-permeable polymers to overcome the problems associated with short post-injection half-life, thereby improving their therapeutic effect [40]. Most delivery systems used for this purpose are hydrophilic networks of polymer chains, known as hydrogels [41]. Hydrogels for muscle regeneration are typically made from natural and synthetic building blocks [42]. They are biocompatible, and exhibit a wide variety of physical properties and biodegradability [43]. Encapsulation of therapeutic proteins into hydrogels as a part of a sustained release strategy has shown improved pharmacokinetic properties of the protein [21, 44]. Such a device may also substantially decrease the overall administered dosage, thus reducing any unwanted side-effects associated with super physiological levels of potent biological molecules in vivo [45–47].

The present study describes the development and characterization of a sustained-release delivery system for recombinantly expressed Cripto in skeletal muscle repair. Recombinantly expressed and purified Cripto protein was prepared using a HEK293 culture system [48]. The purified recombinant Cripto was encapsualted within injectable microspheres made of poly(ethylene glycol)-fibrinogen (PEG-Fibrinogen, PF) [49]. PF hydrogels have been applied for use in tissue engineering and as a controlled delivery system because of their biocompatibility, low toxicity, and biodegradability [50–55]. Furthermore, the PF hydrogels have undergone extensive evaluation to ensure biocompatibility, safety, and efficacy in a clinical study [56, 57]. The PF was prepared into microspheres for minimally invasive injectabilty [58]. A dual photo-initiator emulsion polymerization technique was used to process the PF microspheres containing Cripto [59]. The release of the Cripto was controlled by simple compositional modifications to the PF hydrogel formulation. In vitro experiments were used to verify the stability of the released Cripto for up to 4 weeks and ensure biological activity was not compromised. MRI was used to document the in vivo biodegradation and resorption of the Cripto-laden microspheres in a mouse muscle implantation model. The therapeutic effect of PF-mediated localization and sustained delivery of Cripto in vivo was explored in a cardiotoxin (CTX)-induced tibialis anterior (TA) muscle injury in mice. Histomorphometry of muscle regeneration was correlated with PF biodegradation, myogenic marker Pax7, and differentiation markers eMHC, laminin, and desmin. The results indicate that localized, sustained delivery of Cripto from injectable PF microspheres can help accelerate the repair of damaged muscle tissue following acute injuries.

## Material and Methods

### Synthesis of PEG-diacrylate (PEG-DA) and PEG-fibrinogen (PF)

PEG-diacrylate was synthesized as described elsewhere [60]. Briefly, linear PEG-OH with an average molecular weight of 10 kDa (Fluka, Buchs, Switzerland) was reacted with acryloyl chloride (Merck, Darmstadt, Germany) at a molar ratio of 1.5:1 relative to OH groups in dichloromethane (Aldrich, Sleaze, Germany) and triethylamine (Fluka, Buchs, Switzerland). The final product was precipitated in ice-cold diethyl ether (Frutarom, Haifa, Israel), followed by vacuum drying for 48 hours. The degree of acrylation was quantified by proton-NMR (nuclear magnetic resonance spectroscopy). PEG-fibrinogen (PF) was prepared by conjugating PEG-DA with denatured, reduced fibrinogen chains by Michael-type addition, according to the previously described protocol [55]. Briefly, 7 mg/ml solution of bovine fibrinogen (ID bio, France) in 150 mM PBS containing 8 M Urea was reacted with Tris(2-carboxyethyl) phosphine hydrochloride (TCEP–HCl) (Sigma–Aldrich) at a molar ratio of 1.5:1 to fibrinogen cysteines. Once the protein was dissolved, PEG-DA in a solution of PBS and 8 M urea (280 mg/ml) was added, in a molar ratio of 4:1, to initiate the PEGylation reaction. The reaction was carried out for 3 h at room temperature in the dark. Then, the PEGylated protein was precipitated by adding 4 volumes of acetone (Bio-lab) and was re-dissolved in PBS-Urea to the desired concentration, followed by dialysis (Spectrum 12–14 kDa MW cutoff, USA) against 150 mM PBS for 24 hours at 4 °C. Finally, the fibrinogen concentration in the product was measured by a NanoDrop spectrometer (A-280 nm, PF coeficiency-15.1) and the degree of PEG substitution was calculated according to published protocols [55]. Rheological parameters were calculated using a strain-rate controlled shear rheometer (AR-G2, TA Instruments, Delaware, USA) with a parallel-plate geometry, as detailed below [61].

### Cripto manufacturing and encapsulation in PF microspheres

Recombinant soluble Cripto was produced, purified, and characterized according to protocols described elsewhere [48]. Briefly, the recombinantly expressed Cripto was obtained from stably transfected HEK293 cells expressing a cripto-His- vector (sequence from nucleotide –5 to +156 of the cripto cDNA) [62]. The Cripto-His protein was purified from conditioned medium of the HEK293 cells using the QIAexpress protein purification system (Qiagen, Hilden, Germany). Purified proteins were dialyzed against 50 mM sodium phosphate buffer, pH 8.0. The protein concentration was quantified by a BioRad assay (BioRad Laboratories) and aliquot samples were stored at -80 °C. The Cripto was thawed just prior to use, thus avoiding freeze-thaw cycles of the protein. The Cripto was encapsulated in PF microspheres at a final concentration of 0.16 mg/ml (calculated according to the ratio of 4 µg Cripto per 25 µl injection volume). PF hydrogel microspheres were prepared using a dual-photoinitiator, water-in-oil emulsion method adapted with some modifications from a protocol described by Cohen et al., Pradhan et al. and Franco et al. [54, 59, 63]. Briefly, Cripto protein was encapsulated in the hydrogel precursor solution consisting of 8 mg/ml of PF dissolved in PBS, additional PEG-DA (either 1.5% or 3%, w/v), 0.15% (v/v) triethanolamine (TEOA) as a co-initiator, 1% (w/v) Pluronic F127 as the surfactant solution in PBS and 2.5% (v/v) photoinitiator solution (Irgacure®2959). The additional PEG-DA was used as a crosslinker to increase the crosslinking density of the polymeric network. An emulsion was prepared by adding 200 ml of the aqueous phase suspension to an oil phase solution in a glass test tube consisting of 1 ml mineral oil, 0.15%(v/v) TEOA and 0.5%(v/v) photoinitiator solution (Irgacure® 651). The aqueous and oil phases were mixed, then vortexed (for 4 seconds) and manually rotated (for another 26 seconds) under the exposure of UV light. The emulsion was resuspended in PBS washing buffer and centrifuged at 1200 g for 4 min to separate the aqueous and oil phases. The oil phase was aspirated, and the remaining aqueous phase was resuspended, washed with PBS washing buffer and centrifuged again, to ensure the oil was completely removed.

### PF hydrogel rheological characterization

A strain-rate controlled rheometer (TA Instruments AR-G2, New Castle, DE, USA) was used to measure the shear storage and loss modulus of the hydrogels during the photo-polymerization reaction. The rheometer was outfitted with a parallel-plate geometry and UV curing cell to facilitate the photo-polymerization reaction under oscillatory shear stress. Previous experiments were used to determine the exact frequency (3 rad/s) and strain (2% sinusoidal strain) for performing the time sweep tests [64]. Each material was characterized in triplicate for 5 min, to monitor the photo-polymerization reaction of the hydrogel precursor solution (200 μl). The time sweep test was initiated with a 30 second preconditioning phase, followed by exposure to long-wave UV light (365 nm, ~5 mW/cm^2^) while the storage (G′) and loss (G″) modulus values were continuously recorded with Orchestrator 6.5.8 software. The reported shear storage and loss modulus were taken from the complex shear modulus G* = G′+ iG″ at the conclusion of the test. Two different materials were used in this study to represent the high and low crosslinking density hydrogel formulations. For the low and high crosslinking density formulations, 1.5% and 3% (w/v) additional PEG-DA was added to the PF precursor solution prior to photopolymerization to achieve a final shear storage modulus of approximately G’ = 1.5kPa or G’ = 3.5kPA, respectively.

### Microspheres characterization

The morphology and shape of the PF microspheres were analyzed using an inverted fluorescence microscope (Nikon Eclipse TS100, Nikon, Japan), a digital camera (Digital Sight, Nikon, Japan), and Nikon Nis-Elements F3.00 software (Nikon, Japan). The distribution of Cripto within the microspheres was observed by dual labeling fluorescent markers on the Cripto protein and the PF hydrogel. Cripto was labeled with NHS-fluorescein according to the manufacturer’s instructions (46410, Thermo Fisher Scientific). PF hydrogels were labeled using Rhodamine conjugated PEG-DA that was added to the hydrogel precursor prior to photopolymerization, as described elsewhere [65]. After staining, the PF hydrogel microspheres were imaged using a Zeiss LSM 700 confocal microscope (Carl Zeiss, Oberkochen, Germany). The size distribution of the microspheres was measured using a laser diffraction particle size analyzer (Mastersizer 3000, Malvern Instruments Ltd, Worcestershire, U.K.). Analysis of the size distribution was performed according to protocols described by Cohen et al. [66] Briefly, each particle population was characterized by three parameters: the mean diameter, the uniformity and the span. The uniformity parameter provides information about the absolute deviation from the median particle size. A higher value of uniformity indicates a wider distribution of particle sizes, indicating that the particles vary more in size. The span is another parameter describing distribution spread. Span = (D90 – D10) / D50, where D90, D10, and D50 represent the particle size below which 90%, 10%, and 50% of the material is contained, respectively. A higher span value is associated with a less uniform particle size distribution, while a lower span value indicates a more uniform distribution with less variation in particle sizes. While these two parameters are calculated differently, but both give an indication regarding the size distribution (symmetry of distribution is given by the uniformity value, and width of the distribution is given by the span value).

### In vitro Cripto release

The release of Cripto from the PF microspheres was measured by ELISA as part of the controlled release experimental protocol. Samples of 200 µl PF-microspheres containing 0.16 mg/ml of Cripto were placed in 1 ml of a release buffer made from PBS containing 0.1% (w/v) bovine serum albumin (BSA) and incubated at 37 °C for 27 days on an orbital shaker. At multiple time points, 250 µl aliquots of the release buffer were sampled and the same amount of fresh release buffer was replenished in each tube. At the endpoint of the experiment, the PF microspheres were fully degraded by incubating in an acidic solution (PBS, pH 1.2) for 3 hours and the released Cripto was collected. The cumulative amount of released Cripto from the hydrogels were determined as a function of time using an ELISA kit (R&D systems, DuoSet), according to manufacturer’s instructions. For the total protein released, mouse Cripto antibody (R&D systems) was used for coating the ELISA plate. For the active protein released, a recombinant mouse activin RIB/ALK-4 was used (R&D systems). The signal was detected by Thermo Varioskan^TM^ Spectrophotometer using Skanlt2.2® Software. The percent of Cripto release was calculated at each time point by normalizing the amount of Cripto at each time-point by the total amount of Cripto released throughout the experiment (i.e., the cumulative amount of Cripto released). The encapsulation efficiency was determined based on a calculation using Eq. 1:

1 $${Encapsulation}\,{efficiency}=\frac{{Mass}\,{of}\,{Cripto}\,{loaded}\,{in}\,{the}\,{microsphere}}{{Total}\,{mass}\,{of}\,{Cripto}\,{added}}$$

### In vitro cell viability experiments

The biological activity of the released Cripto was estimated using the indirect transwell method. Briefly, the hydrogels were placed into a 6 well-transwell microplate membrane insert (0.4 μm size exclusion) (Corning, Corning, NY) above the cell surface and submerged in the culture media. Each well was seeded with ~150,000 C2C12 mouse myoblasts that were growing in a medium supplemented with 10% FBS and 2.5 mM HEPES buffer. The treatment groups contained cells in a culture medium with 500 ng/ml of Cripto. The cells were incubated at 37 °C and 5% CO_2_ for 24 hours prior to bioactivity evaluation with Alamar Blue assay or 48 hours prior to evaluation with calcein/ethidium and trypan blue. The C2C12 mouse myoblast viability was investigated by staining the cells with Trypan Blue and counting them with an automated cell counter (Countess®-Invitrogen). Cell morphology and viability was investigated by staining cells with calcein/ethidium according to manufactures’ protocol (Sigma-–Aldrich, Buchs, Switzerland) and then visualizing the cells with a Zeiss LSM 700 confocal microscope (Carl Zeiss, Oberkochen, Germany). Metabolic activity was determined by adding 10% Alamar Blue (AbD Serotec) to the culture medium and measuring the absorbance at 540 and 630 nm using a spectrophotometer (the redox reaction, in which Alamar Blue is reduced by the cells, causes a shift in the spectrometric prolife of the culture solution).

### In vivo biodegradation experiments

To evaluate the in vivo biodegradation and resorption of the implanted materials, the PF microspheres were labelled with a gadolinium (Gd) contrast agent for magnetic resonance imaging (MRI) and implanted in mice according to the experimental plan detailed in Supplementary Figure S1. The labelling of PF with Gd and the calibration of the in vitro MRI signal of the Gd-labelled PF hydrogels was previously described elsewhere [51, 61, 66, 67]. All animal studies were approved by the Animal Board and Safety Committee of the Technion (approval number IL1571119). Six to eight-week-old male C57/Bl6 mice were anesthetized with 0.5–1.5% isoflurane supplemented with oxygen (0.6 l/min). Respiration was monitored during imaging using a respiration monitor (Small Animal Instruments, Stony Brook, New York, NY) and body temperature was maintained using circulating hot water. A muscle injury was induced by injecting 20 µl of 10 µM cardiotoxin (CTX) intramuscularly into the anterior tibialis muscle of the animals and the PF microspheres were injected thirty minutes later. A total of four treatment groups were assessed by MRI (Supplementary Figure S1). The first group was injected with 25 µl of PF microspheres loaded with Cripto without a CTX injury (PF-Cripto); the second group was injected with 25 µl of PF microspheres loaded with Cripto after a CTX injury (Gd-PF-Cripto +CTX); the third group was injected with 25 µl of empty Gd-PF without a CTX injury (Empty Gd-PF); the fourth group was injected with 25 µl of empty Gd-PF after a CTX injury (Empty Gd-PF + CTX). Each treatment group was represented by five treated animals (*n* = 5). All the Cripto treatments contained 25 µl of PF microspheres in PBS with a total Cripto concentration of 4 µg per injection. MRI was performed on a 9.4 T scanner (Bruker Biospec, Ettlingen, Germany), using a transmit/receive cylindrical volume coil (86 mm diameter). MRI was performed at seven time points after the injections. T1-weighted images of the lower body of the mice were acquired using a fast-low angle shot (FLASH) sequence with respiratory gating, 150 µm in-plane resolution, 0.35 mm slice, TR/TE = 5.8/2.7 ms, 10° pulse, FOV = 6 × 4 cm^2^, matrix size = 400 × 266, and 15 repetitions; the total scan time was ~10 min. Data processing was done using Medical Image Processing, Analysis, and Visualization (MIPAV) software (NIH; http://mipav.cit.nih.gov). The 2D images per slices were acquired and the area of Gd-microspheres was manually segmented for calculating the volume and the MR signal-to-noise ratio (SNR) intensity over time. ROIs were later drawn. Both volume and SNR values were normalized to day 0.

### In vivo muscle repair experiments

To evaluate muscle repair following Cripto treatment, PF microspheres were implanted in mice according to the experimental plan detailed in Supplementary Figure S2. All animal studies were approved by the Animal Board and Safety Committee of the Technion (approval number IL1571119). Six to 8-week-old male C57/Bl6 mice were anesthetized by inhalation of 2.5% isoflurane as described above. The muscle injury was induced by injecting 20 µl of 10 µM cardiotoxin (CTX) intramuscularly into the anterior tibialis muscles of the animal (four animals for each treatment). After thirty minutes, the mice were injected in the same area with one of the following treatments: the first group was left untreated; the second group was injected with 25 µl of PF microspheres loaded with Cripto (PF-Cripto), the third group was injected with 25 µl of Cripto in bolus (Bolus-Cripto); the fourth group was injected with 25 µl of empty PF (Empty-PF). The PF-Cripto treatments contained 25 µl of PF microspheres in PBS with a total Cripto concentration of 4 µg per injection; the bolus injection contained a total Cripto concentration of 4 µg per injection in PBS. Mice were sacrificed at days 7 and 23 and the TA muscles were isolated and fixed in 4% paraformaldehyde at 4 °C. Subsequently, muscles were dehydrated in ethanol and embedded in paraffin. Paraffin-embedded muscle samples were cut transversely at a thickness of 8 μm and prepared on slides for histological processing.

### Histology

Paraffin-embedded sections were stained with hematoxylin & eosin (H&E) and assessed by a veterinary pathologist (Patho-Logica, Ness Ziona, Israel). Images of the stained slides were obtained using Olympus microscope (BX60, serial NO. 7D04032) equipped with a digital microscope camera (Olympus DP73, serial NO. OH05504) with an objective providing a magnification of X20. The minimum feret diameter of centrally nucleated myofibers and their percentage were morphometrically analyzed using ImageJ software (National Institutes of Health, USA). The feret diameter is a metric used to quantify the size of individual myofibers in a muscle tissue cross-section. It is defined as the longest distance between two parallel lines drawn perpendicular to the longest axis of the myofiber. This value represents the maximum width of a myofiber. All myofibers within each section were evaluated (at least 1500 cells per muscle section; 16 sections per treatment). Myofibers that contained centrally located nuclei were counted and normalized as a percentage of the total number of myofibers in transvers muscle sections. A quantitative analysis of the extent of edema and the severe inflammatory area within each histological section was also performed using computerized image analysis. This analysis was performed with MATLAB software using color, brightness, distribution, and size properties to distinguish the areas, and to calculate the percent of the inflammatory area and edema area out of the total muscle area.

### Immunohistochemistry

For immunofluorescence (IF) staining, paraffin embedded sections were deparaffinized and rehydrated through washes with xylene and descending grades of isopropyl alcohol. Next, an antigen retrieval (AR) procedure was performed as follows: first, slides were boiled in HIER Citrate Buffer (pH 6.0; Zytomed Systems, Berlin, Germany) for 7 minutes. After boiling, slides were allowed to cool to room temperature and then subjected to enzymatic digestion with 0.4% pepsin solution in 0.01 M hydrochloric acid (HCl) for 1.5 min at 37 °C. This dual-step AR process was necessary to achieve a better signal for double staining with laminin antibody. Slides were rinsed with PBS and blocked with Background Buster solution (Innovex Biosciences, Richmond, CA) for 30 minutes. The sections were then immunoassayed with the following primary antibodies: rabbit anti-laminin (L9393, 1:40; Sigma-Aldrich), rat anti-PEG (1:200; kindly provided by Dr. Steve Roffler, Institute of Biomedical Sciences, Taipei, Taiwan), mouse anti-eMHC (F1.652; 1:45; Developmental Studies Hybridoma Bank), mouse anti-Pax7 (1:10; Developmental Studies Hybridoma Bank), mouse anti-desmin (D1033; 1:40; Sigma-Aldrich). Nuclei were counterstained with DAPI (1:1000; Sigma-Aldrich) and primary antibodies were incubated overnight at 4 °C. After rising with PBS, secondary antibodies were applied at a dilution of 1:200 for 1 hour. The following secondary antibodies were used for detection (1:200; Molecular probe, Invitrogen, UK): donkey-anti-rabbit Alexa Fluro 488, donkey-anti-rabbit Alexa Fluro 647, goat-anti-rat Alexa Fluro 555, donkey-anti-mouse Alexa Fluro 555. Finally, slides were mounted with the anti-fade Fluoromount-G mounting medium (Southern Biotech, 0100-01). Throughout the entire staining process, slides were incubated in a humidity chamber. Stained samples were visualized using 3DHistech Pannoramic 250 Flash III scanner at X20 magnification (3DHISTECH Ltd., Budapest, Hungary). Select regions within each slide were imaged using Zeiss LSM 700 confocal microscope (Carl Zeiss, Oberkochen, Germany). All IF image processing and analysis was performed using ImageJ software. The total area of the PF microspheres was calculated for each tissue section. For quantifying the Pax7-positive cells (Pax7 + ), a counting algorithm was used with DAPI co-localization; cells expressing Pax7 without DAPI co-localization were excluded from the count. For quantifying the positive area of eMHC, images were processed via a threshold tool to segment out and quantify the stained areas. To determine the level of cellular fluorescence of desmin, the corrected total cell fluorescence (CTCF) was calculated according to Eq. 2:

2 $${\rm{Corrected}}\,{\rm{total}}\,{\rm{cell}}\,{\rm{fluorescence}}({\rm{CTCF}})={Integrated\; Density}-({Area\; of\; selected\; cell\; x\; Mean\; fluorescence\; of\; background\; readings})$$

### Statistic

Statistical analysis was summarized using Prism software (GraphPad, Boston, MA, USA). Data from independent experiments were quantified and analyzed for each variable. Each treatment was represented by at least three independent experiments (performed in triplicates), unless stated otherwise. Comparisons between multiple treatments were made by analysis of variance (ANOVA) and a Tukey’s post-hoc test was used to assess the significance of differences between pairs of group means. All data was expressed as mean ± standard deviation (S.D.) or mean ± standard error of the mean (S.E.M.). In all experiments, significant differences were indicated as follows: *p < 0.05, **p < 0.01, ***p < 0.001, and ****p < 0.0001.

## Results

### Rheological characterization of PF Hydrogels

Two formulations of PF hydrogels were characterized by strain-rate controlled rheometry, including compositions with high and low cross-linking density. The PF precursor liquid was supplemented with the addition of PEG-DA (approximately 1.5% and 3%, w/v) for attaining the low and high cross-linking density, respectively. Each formulation was characterized by a strain-rate controlled rheometer (in triplicate) and represented by a time-sweep graph of the shear storage modulus (G’) (Supplementary Figure S3). The average plateau G’ was obtained directly from the time-sweep graphs and used to represent the elastic component of the hydrogel’s mechanical properties. The amount of PEG-DA crosslinker in the formulation significantly affected the plateau G’ of the PF hydrogels, with the two formulations represented by a G’ = 1672 ± 70 Pa and G’ = 3340 ± 253 Pa, respectively.

### Characterization of PF microsphere size and morphology

**Fig. 1:**
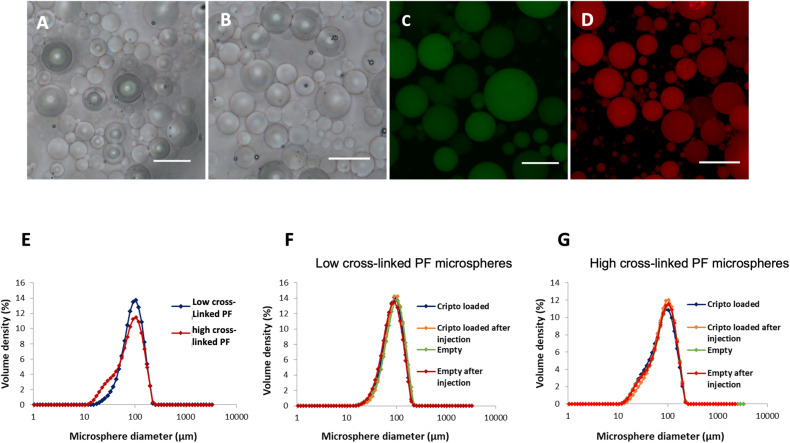
Characterization of PF microspheres. Representative light (**A**, **B**) and confocal (**C**, **D**) images of PF microspheres. **A** Empty PF microspheres made with low cross-link density and (**B**) high cross-link density materials. **C** NHS-fluorescein-labeled Cripto protein, encapsulated within low cross-link density PF microspheres. **D** Empty low cross-link density PF microspheres labeled with rhodamine. Scale bars in all images equals 100 µm. **E–G** Particle size distribution of empty and Cripto-containing PF microspheres. **E** Comparison of the size distribution of empty PF microspheres made with low or high cross-link density materials. The effect of Cripto loading and injection through 30-gauge needle, on size distribution of (**F**) low cross-link density PF microspheres and (**G**) high cross-link density PF microspheres.

The water-in-oil dual photoinitiator emulsion-polymerization method produced Cripto-laden PF microspheres with a spherical shape (Fig. 1A-D) and a diameter with a normal size distribution (Fig. 1E-G). Representative images of the microspheres made with the two PF formulations, based on the low and high cross-linking density compositions, are shown in Fig. 1A-B. Both groups of microspheres exhibited a uniform spherical shape. The introduction of Cripto in the PF formulation, visualized using NHS-fluorescein-labeled Cripto and fluorescence microscopy imaging, did not affect the shape of the microspheres (Fig. 1C). The distribution of Cripto inside the microspheres appeared uniform and homogenous. Empty microspheres made with rhodamine-labeled PF are shown as a control (Fig. 1D). The MasterSizer laser diffraction analysis provided a quantitative measure of size distribution of the PF microspheres, the average size for each microsphere population, the uniformity value, and a span value (Table 1). This size distribution of each population of microspheres was gaussian, with a mean diameter of approximately 90 µm (Fig. 1E). The size data indicated that the PF microspheres with the lower crosslinking density were slightly larger than those with the higher crosslinking density (without Cripto). The low crosslinked PF microspheres have a mean diameter of 90 μm, compared to a diameter of 84 μm for the high crosslink formulation (Table 1). These size distributions were not found to be significantly different from each other (*n* = 3, *p* > 0.05). The addition of Cripto into the PF formulations slightly reduced the average size of the microspheres. In the low crosslinked PF formulation, the Cripto-containing samples have a mean diameter of 96 μm compared to 90 μm without Cripto. For the high crosslinked formulation, the Cripto-containing samples have a mean diameter of 84 μm compared to 80 μm without Cripto. However, these differences were not statistically significant (*n* = 3, *p* > 0.05). The size distributions of the PF microspheres were measured after injection through a 30 G needle. All groups of PF microspheres in the experimental design where unchanged in their size before and after injection through the 30 G needle (Fig. 1F-G).

**Table 1:** Size parameters of PF microspheres.

Sample Name			Mean diameter (µm)	Uniformity	Span
Low crosslinked microspheres (G’ = 1600Pa)	Empty	---	96.7 ± 0.4	0.345 ± 0.0045	1.142 ± 0.0165
	Empty	After injection	87.4 ± 0.3	0.348 ± 0.0013	1.141 ± 0.0031
	Cripto loaded	---	90.9 ± 0.3	0.338 ± 0.0009	1.099 ± 0.0027
	Cripto loaded	After injection	96.3 ± 0.1	0.327 ± 0.0008	1.076 ± 0.0038
High crosslinked microspheres (G’ = 3500 Pa)	Empty	---	88.5 ± 0.6	0.433 ± 0.0011	1.426 ± 0.0029
	Empty	After injection	87.9 ± 0.2	0.426 ± 0.0015	1.405 ± 0.0041
	Cripto loaded	---	84.5 ± 0.2	0.444 ± 0.0016	1.453 ± 0.0057
	Cripto loaded	After injection	91.2 ± 0.3	0.403 ± 0.0026	1.33 ± 0.0086

### Encapsulation efficiency and the release kinetic of Cripto from PF microspheres

**Fig. 2:**
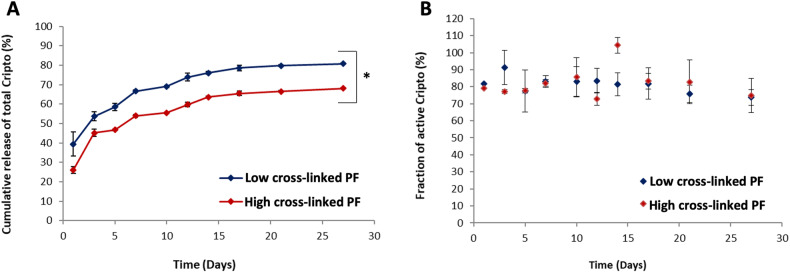
Cumulative release kinetics of total Cripto and the fraction of active Cripto released. **A** Release profile from PF microspheres made with two different shear storage moduli. **B** The percentage of Cripto activity over time. The fraction of active Cripto is calculated by dividing the amount of protein that retained its ability to bind the AlK4 receptor, by the amount of total Cripto in the sample volume. * denotes a statistically significant difference between the two treatments (*p* < 0.05).

The encapsulation efficiency was examined by releasing the entrapped Cripto protein from within the PF microspheres using an acidic solution (pH 1.2). The released Cripto was measured by ELISA and the encapsulation efficiency was then determined by calculating the ratio of Cripto and the theoretical loading percent using Equation 1. The encapsulation efficiency of PF microspheres was in the range between 34-36% (Table 2). There was a small difference in encapsulation efficiency between the low and high cross-linked formulations; however, these results were not found to be statistically significant (*p* > 0.05). The in vitro release profiles of Cripto from PF microspheres were measured in terms of the cumulative release of Cripto into a supernatant liquid over the course of 4 weeks. The release profiles from the PF microspheres, as presented in Fig. 2A, demonstrated a biphasic pattern characterized by an initial burst followed by a sustained phase. During this phase, the Cripto was steadily released with a zero-order kinetic profile. This pattern persisted until the termination of the experiment. The amount of Cripto released from the microspheres was dependent on the shear storage modulus of the PF, with 80% Cripto being released after 27 days from low cross-linked PF microspheres and 68% Cripto being released after 27 days from high cross-linked PF microspheres.

**Table 2:** The mean diameter and Cripto encapsulation efficiency of microspheres made from high or low crosslinked PF formulations.

	Mean dimeter (µm)	Encapsulation efficiency (%)
Low cross-linked PF	96.3 ± 0.1	36.0 ± 3.9
High cross-linked PF	91.2 ± 0.3	34.1 ± 7.4

The release rate of Cripto was measured both in terms of the total protein and bioactive protein, the latter being the ratio between the active protein and the total protein released at each time point (Fig. 2B). The fraction of active Cripto being released was the same for both formulations of PF microspheres. The percentage of released Cripto was determined using the actual amount of protein loaded into the microspheres. The actual amount of Cripto was calculated using the initial loading amount multiplied by the loading efficiency. The Cripto was further verified for stability using SDS-PAGE before and after release from the PF microspheres. The purified Cripto used prior to loading in the PF microsphere treatments was compared to the same Cripto after being release from the microspheres. The Cripto prior to loading was indistinguishable from the Cripto released from low and high cross-linked PF microspheres, respectively (Supplementary Figure S4). The main SDS-PAGE bands corresponding to Cripto have a molecular weight of 27 kDa.

### In vitro biological activity of the released Cripto

We sought to verify that the Cripto retained its bioactivity after being release from the PF microspheres. The bioactivity of Cripto has been linked to regulating satellite cell proliferation and myogenic commitment. To this end, we evaluated the bioactivity of the released Cripto by characterizing its ability to stimulate the proliferation of mouse myoblasts. Accordingly, the Cripto released from both low and high cross-linked PF microsphere formulations was assessed by an indirect transwell co-culture system in which Cripto-releasing hydrogels were placed in the upper chamber and mouse myoblasts were seeded in the lower chamber. The hydrogel volume (and corresponding amount of Cripto) that was added to the transwells was determined based on recombinant Cripto concentrations known to activate primary mouse myoblast proliferation in vitro [24]. We used the in vitro release profiles to estimate the relationship between hydrogel volume and Cripto released into the supernatant to obtain the required Cripto concentration after 48 hours. All treated cell cultures were thus exposed to an equal amount of Cripto (500 ng/ml) by the end of the incubation period. Thereafter, the cells were stained with Calcein/Ethidium for viability, assessed using Alamar Blue reduction for quantifying the metabolic activity, and counted by trypan blue exclusion. The mouse C2C12 myoblast cells treated with Cripto released from PF microspheres appeared more confluent in their culture well plates compared to cells treated with fresh Cripto or untreated cells (Supplementary Figure S5A). Cells treated with both groups of PF-Cripto were elongated, relatively spread, and maintained a characteristic spindled morphology typical of myoblasts. The cell metabolic activity levels of both treated groups were statistically higher compared to the negative control (Supplementary Figure S5B). However, the differences in cross-linking density of the PF release system did not affect the activity of the released Cripto. Incidentally, there was no significant difference observed within the three treated groups in terms of cell viability (Supplementary Figure S5C). Taken together, these data indicate that Cripto activity was maintained in both the low cross-linked and high cross-linked PF hydrogel release systems. Accordingly, the in vivo experiments were performed using the low cross-linked PF formulation.

### In vivo localization and biodegradation of PF microspheres

**Fig. 3:**
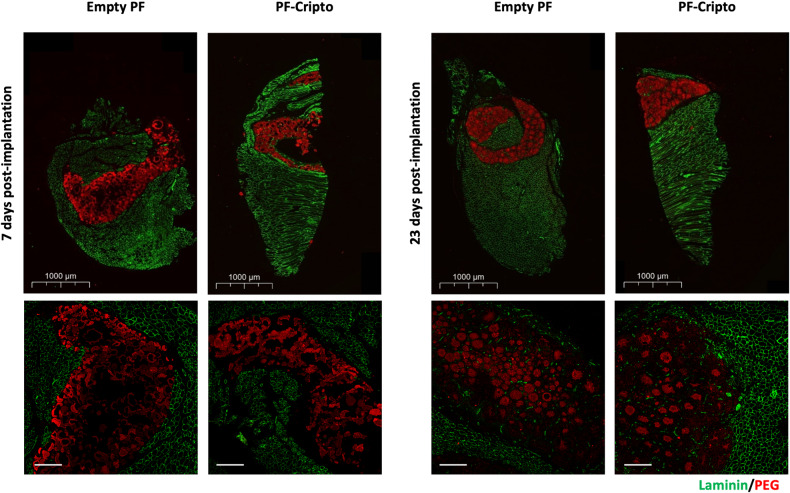
In vivo localization of PF microspheres 7 and 23 days after implantation assessed using histological staining. Immunostaining of the PEG (red) and laminin (green) at 7 days (left) and 23 days (right) post-injection. Top: whole cross-section view (scale bar = 1000 µm), Bottom: high magnification view of region with microspheres (scale bar = 50 µm).

**Fig. 4:**
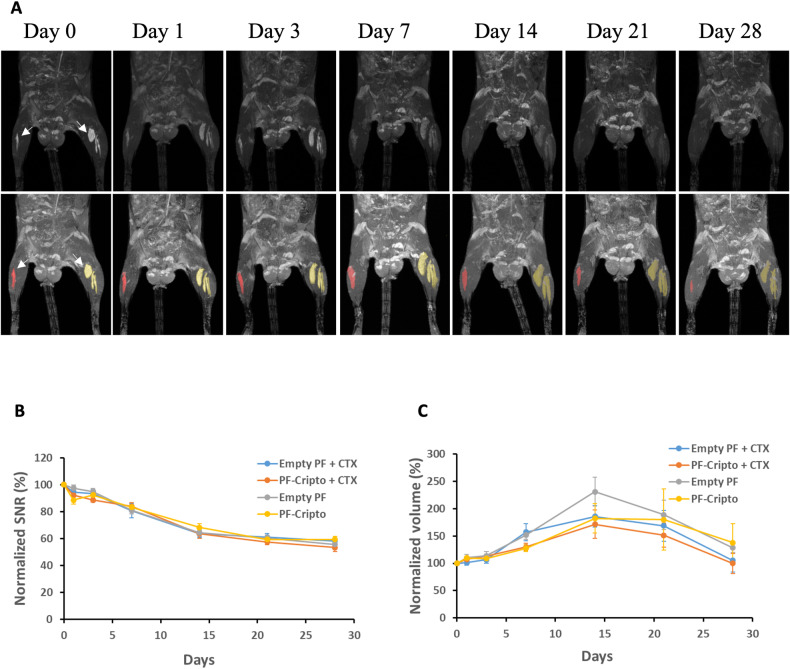
In vivo biodegradation of Cripto-PF microspheres assessed by MRI. The TA muscles of C57-WT mice were injected with 25 µl each of Cripto-Gd-PF or Gd-PF microspheres (with and without cardiotoxin) and imaged using MRI for up to 28 days. **A** Representative MR images of Gd-labelled PF microspheres injected into the TA muscle (arrows show the location of the implant). The left leg was injected with CTX prior to the Gd-PF microsphere injection, whereas the right leg was administrated without CTX. The bottom panel shows the MR images highlighted with false colors to designate the region of the implant in the CTX-treated muscle (yellow) and in the uninjured muscle (red). **B** Graphical representation of the segmentation data reveals a reduction in the transient Signal-to-noise (SNR) parameter. **C** The implant volume parameter (normalized to day 0) exhibits an initial increase followed by a decrease. Data in **B** and **C** is presented as mean ± SEM; *n* = 5 mice per group.

After injecting the PF into the TA muscle, immunofluorescence staining of PEG and laminin helped to identify the placement of the microspheres within the muscle tissue. Figure 3 shows the PEG-based microspheres localized within the TA muscle after 7 and 23 days. The implanted material was distributed mostly around the injection site and did not appear to diffuse throughout the muscle. At day 7 after injection, the microspheres were more densely packed in the muscle as compared to day 23 after injection. The microspheres maintained their spherical shape, even after 23 days. The implant localization and biodegradation were further assessed using MRI analysis of PF microspheres. The local in vivo biodegradation of the PF microspheres was evaluated for four different treatment conditions as illustrated in Supplementary Figure S1. Throughout the follow-up period of 28 days, the labelled PF microspheres remained at the site of injection in all four treatment groups (Fig. 4A and Supplementary Figure S6). The gradual reduction in MR signal intensity (expressed as normalized SNR) was observed over time for each treated group (Fig. 4B). The normalized microsphere volume was also assessed and showed an initial increase followed by a gradual decrease after 14 days post-injection (Fig. 4C). At Day 7, the volume of the PF-Cripto treatments was statistically lower than the volume of the PF-only treatments (*n* = 5, *p* < 0.05). The CTX also reduced the normalized volume of the implants, particularly after day 14. Taken together, these data indicated that both the CTX injection and the presence of Cripto reduced hydrogel dispersion.

### In vivo biocompatibility analysis

In vivo biocompatibility of the PF implants was assessed by measuring the areas of tissue inflammation and edema (Supplementary Figure S7). In terms of quantitative inflammation, the empty PF group at day 7 had the highest response and second highest response was in the PF+Cripto group (Supplementary Figure S8A). The differences between these groups were significant (*p* < 0.01). After 23 days, the empty PF group decreased and displayed similar inflammation to the PF+Cripto group (*p* > 0.05). No inflammation was observed in the WT group (Supplementary Figure S7C). In terms of Edema, the results in the non-WT groups were similar to the results in the WT group (*p* > 0.05) (data not shown). In the non-WT animals at day 7, the results were highest in the Empty PF group and lowest in the bolus Cripto group (8.91%) (Supplementary Figure S8B). The differences between these groups were significant (*p* < 0.01). At day 23, the results were lower than at day 7. At day 23, the results were highest in the untreated group (~8.84%), and lowest in the bolus Cripto group (6.34%) (Supplementary Figure S8B).

### Skeletal muscle regeneration induced by PF-Cripto

**Fig. 5:**
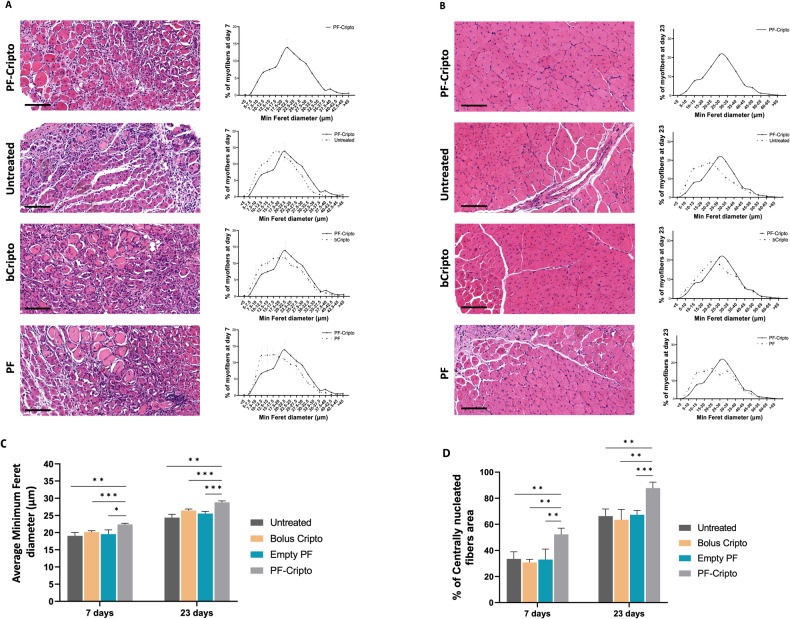
Histological assessment of muscle regeneration over the period of 7 and 23 days after CTX injury and treatment with and without controlled release Cripto. Representative H&E images of TA muscle cross sections at 7-days (**A**) and 23-days (**B**) post-CTX injury and treatment. The histograms of the minimum feret diameter values represent the size distribution of the myofibers in each treated muscle (always compared to PF-Cripto). Quantitative analysis of average minimum feret diameter (**C**) and percentage of centrally nucleated fiber area (**D**) shows a trend towards statistically higher values in the PF-Cripto treatment. Data is presented as mean ± SD; *n* = 4 mice per group; ***P < 0.001, **P < 0.002, *P < 0.03 compared to PF-Cripto treated mice. Scale bar 100 µm.

Skeletal muscle regeneration following acute CTX-induced muscle injury was histologically assessed in four treatments according to the study design illustrated Supplementary Figure S2. Histological cross-sections of TA muscle stained with hematoxylin and eosin (H&E) showed the typical morphology of muscle injury after 7 days post-CTX. All four treatment groups displayed typical patterns of regeneration, including an abundance of small, newly formed myofibers with central nuclei that replace the damaged myofibers (Fig. 5A). By day 23 postinjury, a substantial recovery of muscle architecture was observed in all treatment groups (Fig. 5B). The muscle damage, which was assessed for regeneration using histological morphometric analysis for centrally localized nuclei and minimum feret diameter, was quantitatively represented with histograms. The histograms for PF-Cripto and the other treatments are shown in Fig. 5A-B. The histograms of PF-Cripto relative to the other treatments show a slight shift towards larger diameters at day 7, and a more pronounced shift towards larger diameters at day 23. Although muscle regeneration was apparent in all groups, muscles treated with PF-Cripto demonstrated greater improvements, which manifested in a more robust regenerative response of the TA muscle.

To further support these observations, we undertook a quantitative analysis to reveal that the proportion of myofibers with centrally located nuclei in muscles injected with PF-Cripto was significantly higher in comparison to the three other groups at both time points (Fig. 5C). To complement these findings, morphometric analysis was applied to determine the minimum feret diameter of myofibers with central nuclei. For all treatment groups, myofibers progressively increased in diameter with time following injury and treatment administration (Fig. 5D). By the seventh day post injury, the average minimum feret diameter of the myofibers from muscles treated with PF-Cripto had significantly increased compared to the control groups (*p* < 0.05). These results are consistent with the shift in myofiber size distribution toward larger diameter fibers in the PF-Cripto treatment (Fig. 5A). Comparable results were also obtained for all treatment groups at the 23-day time-point (Fig. 5B).

### Expression of myogenic markers in TA muscle treated with PF-Cripto

**Fig. 6:**
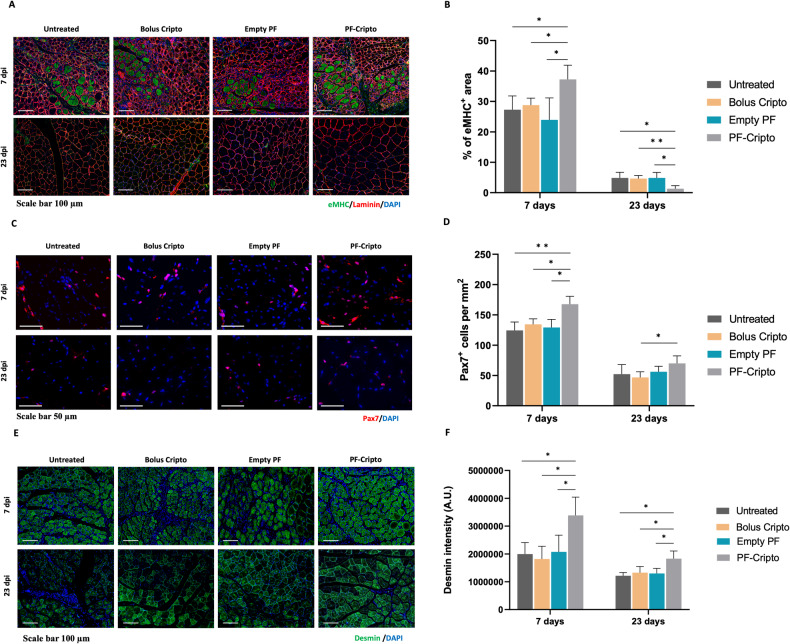
Expression of markers associated with skeletal muscle regeneration over the period of 7 and 23 days following CTX injury and treatment with and without controlled release Cripto. Immunostaining of markers was performed on CTX-induced TA muscles either untreated or treated with bolus Cripto, empty Cripto or PF-Cripto after 7 and 23 days. Representative images and corresponding marker-specific quantifications are shown for: **A-B** Laminin (red) and eMHC (green) (Scale bars = 100 μm); **C-D** Pax7 (red) (Scale bars = 50 μm); and **E-F** Desmin (green) (Scale bars = 100 μm). Data is presented as mean ± SD; *n* = 4 mice per group; **P < 0.002, *P < 0.03 compared to PF-Cripto treated mice.

To further confirm the enhanced muscle regeneration mediated by the controlled release of Cripto from the PF microspheres, we performed immunofluorescence staining for Pax7, eMHC, desmin and laminin. The eMHC staining revealed a typical pattern of elevated expression one week following CTX injury, which was subsided by day 23 (Fig. 6A). At day 7, the percentage of eMHC-positive area was significantly higher for muscles treated with PF-Cripto than for the other control groups (Fig. 6B). By day 23 post-CTX injury, the muscle regeneration as indicated by eMHC staining had proceeded nearly to completion in all treatment groups. The percent of eMHC-positive area in the PF-Cripto treated group at day 23 was significantly lower when compared to controls (Fig. 6B). Pax7-positive cells were evaluated in each group as a measure of the satellite cell population within the muscle after injury and treatment. The Pax7 staining revealed elevated numbers of Pax7-positive cells one week following CTX injury, which subsided by day 23 (Fig. 6C). At day 7, muscles treated with PF-Cripto showed the highest percentage of Pax7-positive cells (*p* < 0.05) (Fig. 6D). Notably, after 23 days, when regeneration was near completion, the basal pool of Pax7-positive cells was higher in PF-Cripto treated mice compared to all other treatments (Fig. 6D). To gain additional insight into the overall pro-regenerative effect of PF-Cripto, we examined the expression of desmin (Fig. 6E). The desmin was evident in all groups at day 7 and day 23. In all experimental groups, desmin intensity was significantly elevated at day 7 compared with day 23 (Fig. 6F). However, mice treated with PF-Cripto had markedly higher intensities of intracellular expression of desmin after 7 days, which remained robust even after 23 days. Laminin was present early after the injury at day 7 in all CTX-treated groups, with its characteristic morphology associated with early-stage muscle repair [68]. By day 23, laminin expression was consistent with more mature myofibers, signifying the near completion structural repair process.

## Discussion

### Designing a Hydrogel Carrier for Cripto

The Cripto protein required a method of delivery that localized the protein to the muscle injury, protected the protein from degradation, and provided sustained delivery to the regenerating muscle tissue. Accordingly, a microcarrier delivery system was developed for intramuscular injection of Cripto to provide stability after in vivo placement. For their in vivo administration, the injectable PF microcarriers also needed to be small enough to easily pass through a 30-Gauge (30 G) needle. There are several methods used to produce hydrogel microcarriers in the form of microspheres that are sufficiently small to enable such injectability, including microfluidic polymerization, emulsion-polymerization, and drop-wise polymerization [69]. In this study, the PF microspheres were fabricated using a water-in-oil emulsion-polymerization method, which has previously been shown to efficiently encapsulate mRNA and viable cells in PF microspheres [59, 66, 70, 71] The estimated dimensions of the microspheres, in the range of 100 micrometers, were compatible with intramuscular administration procedures [72]. Although the dual-photoinitiator aqueous-oil emulsion polymerization technique worked well for producing very small PF microspheres, there are several limitations to this approach. For example, the use of an oil phase requires extensive washings to eliminate it prior to in vivo administration. Drop-wise polymerization techniques that do not use an oil phase can overcome this limitation; however, these techniques are inherently rate-limiting and difficult to scale for mass production [73]. In contrast, the emulsion polymerization procedure is highly effective when bioprocessing large volumes of microspheres in less than a minute. This bioprocessing is scalable and highly compatible when encapsulating water-soluble proteins in the PF. It is also very difficult to obtain very small microspheres using drop-wise techniques, thus limiting the surface-area-to-volume ratio and reducing the efficient diffusion of therapeutic protein to the tissue upon implantation. In our study, the emulsion polymerization technique succeeded in creating sufficiently small microspheres for injectability, but the polydispersity of the microsphere population could pose a challenge for achieving a more uniform release profile from the system. Microfluidic-based techniques can be employed to overcome the limitations of high polydispersity, but challenges of scale-up and mass production have complicated the translation of microfluidic systems for microsphere bioprocessing [74]. Therefore, we opted to use the emulsion polymerization technique – despite its limitations – when producing the Cripto-laden PF hydrogels for our in vivo experiments.

To engineer the Cripto-releasing microspheres, we placed Cripto in PF hydrogel precursor solution prior to emulsion photo-polymerization, thereby entrapping the Cripto in the gel. We further tested how our different formulations, and presence of Cripto, affected the size of the microspheres and showed this to be negligible (Table 1). Specifically, the PF microspheres with the lower modulus were only slightly larger than those with the higher modulus. This result may be attributed to the fact that the higher modulus hydrogels undergo less swelling because they exhibited a higher degree of crosslinking owing to their increased PEG-DA concentration. Importantly, there was no difference in the PF microsphere size distribution after passing through a 30 G needle for either formulation, thus verifying that shape integrity was maintained. This result underscores the ability of all groups of PF microspheres to withstand the high shear stresses associated with passing through a small-gauge needle, without breaking apart during the injection. This is an important attribute when designing an injectable pre-polymerized controlled-release hydrogel system, where shape and size can alter the release kinetics of the payload.

Characterizing the encapsulation efficiency and distribution of the Cripto protein inside the microspheres is also of particular importance because these factors can readily affect the Cripto release characteristics [75]. The fluorescence images of the PF microspheres show a uniform and homogenous distribution of Cripto (green) within the microspheres, confirming the emulsion polymerization technique is effective for encapsulating the protein within the gel network. In terms of the encapsulation, the results indicated a low efficiency (<40%) associated with this technique (Table 2). One possible explanation for the poor loading efficiency is due to the potential Cripto losses during multiple washing steps needed to remove the oil phase during the microsphere preparation procedure, where the burst release phase is likely to take place. Previous studies performed in our laboratory demonstrated a direct correlation between the washing step and the loss of Cripto protein (data not shown). Further compounding these losses could be the high surface area to volume ratio of the micrometer-scale microspheres, which could lead to much of the entrapped Cripto protein being released during the burst-release phase. Specifically, the Cripto, which may be either loosely adsorbed on the surface or absorbed within the core of the microspheres, can more easily diffuse to the external medium due to the shorter migration pathway. Others have also confirmed this, showing that the amount of protein released during or immediately after crosslinking was removed together with the oil phase throughout the washing process [76–78]. The encapsulation efficiency was not significantly affected by the crosslinking density, as indicated by the negligible differences observed for the formulations of PF hydrogels made with a low and high modulus. To overcome the low loading efficiency of the emulsion polymerization technique, we loaded much higher concentrations of Cripto to the precursor solution prior to polymerization so that after washing, we would be left with 4 µg Cripto per injection, as per our delivery target for the in vivo studies. In terms of in vitro biocompatibility, we cultured mouse myoblasts in the presence of PF-Cripto microspheres and quantified cell metabolism and cell viability compared to free Cripto and empty controls (Supplementary Figure S5). All treated groups demonstrated high viability and high metabolism of the mouse myoblasts, pointing to good biocompatibility of the CP-Cripto microspheres.

### Controlling the Release Rate of Cripto

The controlled release feature of the PF microspheres was based on diffusion of the protein from the hydrogel after the photopolymerization. The cross-linking density of the PF network affected the diffusion of Cripto from the hydrogel and altered the Cripto release properties. Consequently, the crosslinking density was augmented by adding additional PEG-DA to the polymer network [79], and the hydrogel shear storage modulus was used to verify that the degree of crosslinking of the hydrogels was affected by the two different concentrations of the PEG-DA crosslinker. Kopač et al. showed that this is a reliable correlative measure of hydrogel crosslinking density in polysaccharide-based hydrogels [80]. The two formulations in this study used different concentrations of additional PEG-DA crosslinker to alter the crosslink density to affect the mesh size of the network and reduce the diffusion of the Cripto from the microspheres. As expected, the two hydrogel formulations resulted in different release profiles of the Cripto, as shown in our in vitro controlled release study. One of the limitations of this approach is that the PEG in our material system controlled both the mesh size of the network and the biodegradation of the hydrogel, thereby altering the Cripto release through two mechanisms [53]. However, our previous studies have demonstrated a clear correlation between the diffusivity of proteins from PF hydrogels and the molecular weight of the added PEG, suggesting that protein release can still be tightly be regulated by a combination of both degradation and diffusion [81, 82]. Others have also shown that the mesh size of PEG-based hydrogels, when controlled by the molecular weight of the PEG, can confine the diffusion of proteins, thereby affecting the release of the entrapped molecules [83, 84]. It is important to note that it is difficult to correlate between our diffusion-based in vitro Cripto release measurements and the actual in vivo Cripto delivery. We presume that the in vivo Cripto release is mediated both by diffusion and by the biodegradation of the PF microspheres. We opted to use the low crosslinked formulation because it ensured that nearly 80% of the entrapped Cripto would be liberated by diffusion after four weeks, and the in vivo release rate is likely faster than that, given that biodegradation facilitates further liberation of Cripto from the gels.

For the release measurements, we used an assay that is premised on well-established protocols to characterize the diffusion of Cripto protein from the PF hydrogel to the supernatant during a 24-day experimental duration [85, 86]. The release assay involved periodic collection of samples and replenishment of fresh aqueous supernatant to maintain a constant volume. One limitation of this approach is that the replenishment of the supernatant with the fresh solution can alter the diffusion kinetics by periodically altering the amount of Cripto outside of the microspheres. This can be avoided by using very small sampling volumes of collected supernatant or few collection points to ensure that the sampling volume does not significantly deplete the total supernatant volume. Nevertheless, in our experiments, multiple collection points were required, and the volume of the collected supernatant needed to perform the ELISA assay was large relative to the total supernatant volume. Consequently, all the collected samples were stored frozen and analyzed collectively by ELISA at the end of the release experiment to minimize loss of activity during storage. The activity of the Cripto was measured with both ELISA assay using an antibody that recognizes the active site of the protein, as well as by evaluating the protein using SDS-PAGE. The SDS-PAGE results confirm the stability of Cripto during the in vitro release and demonstrate that the released proteins did not undergo significant degradation or fragmentation during the encapsulation process and remained intact upon release. In the SDS-PAGE results, a band at 70 kDa appears in all lanes; this is likely to be the residues of fibrinogen chains derived from the PF microspheres after extracting the Cripto. We have previously shown by SDS-PAGE that PF appears similar to denatured fibrinogen when released from the hydrogel [87]. Consequently, one cannot exclude the potential biological effects of this released fibrinogen from the PF on the metabolic activity of the cells in our in vitro or in vivo studies. Aside from the likely PF band, there were no additional bands in the SDS-PAGE results to indicate the presence of Cripto aggregates or fragments larger or smaller than the expected molecular weight of Cripto.

### In vivo Safety and Biodegradation of Cripto-Bearing Microspheres

The PF hydrogel has also undergone extensive clinical evaluation to ensure its safety and biocompatibility [56, 57]. Nevertheless, the in vivo biocompatibility of the PF-Cripto and PF materials was further assessed in the muscle injury using histomorphology measurements of inflammation and edema in the tissue sections (Supplementary Figure S7-S8). The pro-inflammatory response to PF was high at day 7 and subsided by day 23. Interestingly, the PF-Cripto treatment mitigated the inflammatory response to PF at day 7, supporting the hypothesis that Cripto is involved in moderating muscle inflammation [37]. The pro-inflammatory response to PF has been well documented by our group in several past publications [52, 53, 88]. We speculate that the sustained mild, yet persistent inflammation caused by the residual PF present in the tissue ultimately contributes to natural tissue repair pathways [88]. We have shown in other studies that once the PF is fully resorbed, the pro-inflammatory effect subsides, and tissue homeostasis is restored [52].

An in vivo study was conducted to determine the biodegradation rate of the PF-Cripto microspheres, and the ability of the PF-Cripto treatment to accelerate the regeneration of skeletal muscle after an acute injury induced by cardiotoxin (CTX). An injury to the TA muscle of mice was introduced via intramuscular injection of CTX. After a 30-minute delay, the muscle was treated with the therapeutic Cripto. The delay in administering the treatment was designed to enable the CTX to diffuse throughout the muscle and create a uniform injury in the affected muscle [89, 90]. The treatment groups included a PF-Cripto implant, and empty PF implant and bolus Cripto injection. An additional group was left untreated as a control. All mice were sacrificed 7- and 23-days postinjury. When the PF implant was injected in vivo, the histological data indicated good biocompatibility as well as a reduction in the implant size. This outcome, which is suggestive of biodegradation of the microspheres, also underscores that implant resorption was not altered by the presence of the Cripto. However, it is extremely difficult to assess implant volume using histology alone.

A more quantitative result achieved by the MRI methodology using SNR and an implant volume metric helped resolve the biodegradation kinetics of the implants in our study. For this purpose, PF microspheres were labeled with the contrast agent gadolinium (Gd) and were injected intramuscularly into the TA muscle. The MRI analysis combined with our Gd-labeling technique have been previously used to non-invasively track PF implants after subcutaneous implantations [51, 67] and intramuscular injections [66]. Two factors were examined in the MRI biodegradation experiments: with and without Cripto, as well as with and without CTX. A progressive decrease in the SNR over time was demonstrated, irrespective of the treatment, confirming that the PF was being gradually degraded. The reduction in SNR is associated with the resorption of the microspheres in the muscle tissue [66]. However, when evaluating the implant volume, a different trend was apparent. The volume was initially increased and subsequently began to reduce after 14 days. The volume can be a measure of the dispersion of the hydrogel within the muscle tissue after implantation [66]. This difference in trends between the SNR and volume data is attributed to the methodology of quantification. Whereas the values of SNR represent the implanted PF concentration within the tissue, the values of volume can indicate swelling, biodegradation, and dispersion of the PF microspheres within the tissue [91, 92]. We speculate that these results suggest that the initial increase in volume is related to the swelling of the microspheres after their placement in the tissue and the dispersion of the microspheres around the area of injection. Moreover, the constant decrease in PF concentration (i.e., SNR) highlights the efficient biodegradation of the implant in the tissue.

### In vivo muscle repair with Cripto-bearing microgel treatment

To assess the extent of muscle repair in the CTX model, we evaluated a number of parameters, including the percentage of centrally nucleated myofibers and their size; both these parameters are indicative of hallmarks of the muscle regeneration process. One of the most pronounced hallmarks of regenerating myofibers following CTX muscle injury is the number of centrally localized nuclei [93, 94]. We analyzed the minimum feret diameter of centrally nucleated myofibers, as well as their proportion of the total myofibers in all the experimental groups. In our results, the increase in myofiber size, accompanied by the increased percentage of regenerating myofibers (i.e., those with central nuclei), indicates that the extent of regeneration in the CTX-induced mice was significantly enhanced when the injury was treated with PF-Cripto. Specific markers that were evaluated for indications of muscle repair include the eMHC. This is a developmental isoform of myosin heavy chain that is expressed in actively regenerating muscle fibers. As the new fibers mature, eMHC expression is downregulated and replaced by adult myosin heavy chain isoform [95]. The percentage of eMHC-positive cells within PF-Cripto treatment was the highest at day 7 but the lowest at day 23, indicating that the regeneration proceeded faster with the PF-Cripto treatment compared with the other control groups and the muscle was almost fully repaired. Others have also shown that eMHC is expressed in actively regenerating muscle fibers and downregulated as maturation is reached [96].

The population of satellite cells, which is known to play a major role in facilitating muscle regeneration, was evaluated by the expression of Pax7. In healthy muscle, satellite cells remain quiescent, but when the muscle is damaged, these cells are rapidly activated to produce a pool of proliferating myoblasts that will eventually differentiate and fuse to repair and replace the damaged myofibers [97]. The paired box transcription factor Pax7 is expressed in quiescent and activated muscle satellite cells, but it is downregulated as myogenic differentiation advances [98]. Others have used the marker for Pax7 to identify MuSCs present in the muscle fiber that can be available for regeneration [96]. They have shown that higher Pax7 staining indicates more MuSCs are being stimulated because of the CTX injury and favorable regenerative conditions. In our study, the higher number of Pax7-positive MuSCs observed in the PF-Cripto-treated group was another indicator of the enhanced regeneration capacity achieved by the treatment. Another myogenic marker, desmin, provides insight about the quality of the regenerating muscle fiber, in terms of structural integrity and function of muscle [99]. Desmin is an intermediate filament protein which is expressed in newly formed myofibers, and its expression continues into the mature myofiber (where it is pivotal for maintenance of mature myofiber integrity). Others have shown that high levels of desmin expressed at the latest stages of muscle repair are correlated with more stabilized mature myofibers [96]. The Desmin staining results in our study suggest that the high levels of desmin expressed after 23 days in the PF-Cripto-treated muscles is correlated with more stabilized mature myofibers in the later stages of regeneration. Laminin expression within the muscle injury was used to assess the structural aspect of the repair. Laminin is a key component of the extracellular matrix in regenerating skeletal muscle, playing a central role in muscle cell adhesion and differentiation [68]. In all treatments, the laminin expression pattern was consistent with the expected trajectory of an acute CTX muscle injury. At day 7, the laminin morphology revealed an active structural repair processes underway within the muscle tissue, whereas by day 23, the laminin expression indicated myofiber maturation and a structurally mature morphology in all treatments. Given these collective findings, it is likely that the increased expression of myogenic markers, as well as the improved histological morphology of the injured muscle, confirm that PF-Cripto-mediated therapy enables substantial enhancement of muscle regeneration following injury. Consequently, the quantitative immunofluorescence data that was used to assess tissue repair was not substantiated by other techniques (e.g., western blotting or PCR), this being a limitation of the current study.

Although the enhancement of muscle repair in the PF-Cripto treatment was statistically significant compared to all other treatment conditions, the extent of measured improvement at day 23 was modest. This is most likely because of limitations of the CTX injury model, namely the acute nature of the injury and its high capacity for self-repair. Other traumatic muscle injury models have been explored, including crushing and ablation models, yet the CTX model is often used to study the mechanism of muscle repair because of the minimal invasiveness and reproducibility [100]. Besides minimizing the risk of infection associated with surgical intervention, the CTX injury produces a uniform wound, without a major traumatic component, leading to consistent healing patterns. Nevertheless, the repair process following CTX is very complex, involving activation of muscle satellite cells, changes in protein expression, clearance of necrotic fiber debris, macrophage phenotype conversion, proliferation, and differentiation of myoblasts as well as fusion, fibrosis, and calcification [100]. And while all these processes can influence the final regenerative outcome, the reproducibility of the CTX wound healing still allows us to quantitatively assess effects of Cripto release using simple quantitative measures such as minimum feret diameter and histomorphometry – outcomes that could be prone to higher variability with more aggressive tissue injury models.

To overcome the problems associated with the inherent self-repair in the CTX model, we evaluated at days 7 and 23 to capture both the early-phase response to treatment and longer-term effects in the acute injury phase. Collectively, these two data point provided us with important information about how our different treatments affected the healing kinetics in the acute injury. The day 7 follow-up was used to assess the initial tissue reaction to the Cripto treatment relative to the other groups, including key morphometric makers of repair such as eMHC, Pax7 and desmin. The day 23 data provide insight into the extended impact of the treatment on myofiber maturation, including laminin expression, minimum feret diameter and centrally nucleated fiber area. Consequently, the enhanced indicators of muscle repair associated with the PF-Cripto treatment at day 23 suggests that one can accelerate the kinetics of repair through Cripto-mediated events observed at day 7. Interestingly, Cripto administrated in bolus form was not sufficiently potent to illicit the same cellular response at day 7 as the PF-Cripto, despite both treatments having the same dose. Consequently, the day 23 data for the bolus-Cripto treatment was statistically similar to untreated control, signifying the cruciality of the sustained delivery of Cripto to a muscle injury for augmenting the natural repair mechanisms. In this context, we also cannot exclude the potential synergistic effects between the Cripto and the released fibrinogen from the PF on the repair processes; this being a limitation of the present study. Nevertheless, our finding suggests that the therapeutic potential of protein molecules that target the natural muscle repair pathway may be realized only when using a sustained release approach. Others have reached similar conclusions with other bioactive molecules used in muscle repair, including myostatin inhibitors. Estrellas et al., using an injectable hydrogel delivery system made from hyaluronic acid and processed skeletal muscle extracellular matrix, delivered the myostatin inhibitor RK35 in a Duchene muscular dystrophy (DMD) mouse model (MDX) [21]. They showed that injecting MDX mice muscles with RK35 alone produced similar outcomes to saline controls, whereas hydrogel injections containing RK35 stimulated increased muscle mass, larger cross-sectional area, and higher levels of eMHC. These types of studies underscore the necessity of integrating hydrogel delivery and myogenic therapeutics toward realizing the full potential of such an approach in muscle repair. Further studies using different muscle injury models, such as the volumetric muscle loss (VML) model [101], or using other muscle repair antagonists, such as Follistatin [22], will help clarify the extent to which a hydrogel-based approach could be clinically beneficial.

## Conclusions

The treatment of an acute muscle injury using exogenous recombinant Cripto protein with controlled release biodegradable PEG-fibrinogen (PF) hydrogel microspheres was evaluated. The dual photoinitiator emulsification procedure used for making the microspheres was able to produce PF microspheres with dimension that are compatible with injection through a 30-gauge syringe. The Cripto protein was entrapped within the PF microspheres and released for several weeks by diffusion or liberated from the microspheres as a result of diffusion and hydrogel biodegradation. The encapsulation proved effective in providing a stabilizing effect for the Cripto, as indicated by the sustained protein activity after its release from the microspheres after 28 days. The in vivo localization and biodegradation of the PF-Cripto microspheres in the target muscle was confirmed for up to 4 weeks by MRI analysis. The combined effects of localized Cripto delivery, sustained Cripto activity and controlled Cripto release into the target muscle over 3-weeks provided enhanced muscle regeneration following acute cardiotoxin injury in the tibialis anterior muscles of a mouse muscle repair model. The PF-Cripto-treated muscle injuries showed higher levels of muscle progenitor cells present early in the process, and better muscle repair indicators after three weeks of regeneration, including histomorphometry and biomolecular markers. The bolus Cripto was unable to provide augmented muscle repair in the CTX injury, suggesting that sustained delivery of Cripto to the muscle injury may be required to realize its full therapeutic potential. Accordingly, better delivery of the therapeutic proteins may provide the conditions required for stimulating muscle repair through muscle stem cell activation in difficult-to-treat muscle injuries or in certain muscle disease.

### Supplementary information

Supplementary Figure Legends

Supplementary Figures

## Data Availability

Upon request, the authors will provide datasets that are not presented in the main manuscript or additional supporting files.
