# Supplementary material for: Development of a local controlled release system for therapeutic proteins in the treatment of skeletal muscle injuries and diseases
Source: Cell Death Dis. 2024 Jul 2;15(7):470. doi: 10.1038/s41419-024-06645-2 (PMC11219926; doi:10.1038/s41419-024-06645-2)
Supplement: Supplementary file 1 — Supplementary Figure Legends [file 41419_2024_6645_MOESM1_ESM.docx]

**Supplementary Figure Legends**

**Supplementary Figure S1: Illustration of the in vivo biodegradation experimental design.** The TA muscles of C57-WT mice were injected with 25 µl each of Gd-PF+Cripto or Gd-PF microspheres (with or without 20 µl of cardiotoxin, CTX). MRI assessments were performed on day 0, day 1, day 4, day 7, day 14, day 21, day 28. Animals were sacrificed at day 28.

**Supplementary Figure S2: Illustration of the in vivo muscle regeneration experimental design.** The TA muscles of C57-WT mice were injected with 20 µl of cardiotoxin, CTX, followed by 25 µl each of PF+Cripto, Bolus Cripto, or PF microspheres (or empty control). Animals were sacrificed at day 7 and day 23. Histological assessments were performed with H&E and immunofluorescence for eMHC, laminin, desmin, Pax7.

**Supplementary Figure S3: Rheological time sweep graphs of the shear storage modulus of PF hydrogels.** Two PF formulations were prepared and tested by exposing the liquid precursor to UV light activation after 60 seconds (indicated on the graph by the red arrow). The plateau shear storage modulus, G’(Pa), was significantly higher for the high crosslinked formulation as compared to the low crosslinked formulation.

**Supplementary Figure S4: SDS-PAGE analysis of the stability of Cripto released from PF microspheres.** Lanes A and B present two separate fresh batches of Cripto which were not entrapped in the PF microspheres. Lanes C and D represent Cripto which was released from the PF microspheres made from the low crosslinked and high crosslinked formulations, respectively. The molecular weight ladder (left most lane) underscores the location of a marker protein with a MW of 25 kDa and a different marker protein with a MW of 70 kDa.

**Supplementary Figure S5: Biological activity of the released Cripto.** (A) Live/dead asssay results and fluorescent images of C2C12 myoblasts under different treatments, where calcein (green) represents live cells, and ethidium (red) represents dead cells. Scale bar = 100µm. (B) Cell metabolic activity as detemined by Alamar Blue assay, where activity is expressed as fold-change over control. (C) Quantitative cell viability of C2C12 myoblasts under different treatments as measured by a Trypan Blue assay. Data is presented as mean ± SD; n >3 per group; ****P < 0.0001, ***P < 0.001, **P < 0.002, *P <0.03 compared to untreated samples.

**Supplementary Figure S6: Additional representative data from in vivo biodegradation of Cripto-PF microspheres as assessed by MRI.** The TA muscles of C57-WT mice were injected with 25 µl each of Cripto-Gd-PF or Gd-PF microspheres (with and without cardiotoxin) and imaged using MRI for up to 28 days. (A) Representative MR images of Gd-labelled PF microspheres injected into the TA muscle (arrows show the location of the implant). The left leg was injected with CTX prior to the Gd-PF microsphere injection, whereas the right leg was administrated without CTX. The bottom panel shows the MR images highlighted with false colors to designate the region of the implant in the CTX-treated muscle (yellow) and in the uninjured muscle (red).

**Supplementary Figure S7. Representative histopathological photographs.** Histological sections are stained with H&E and analyzed with MATLAB software, using color, brightness, distribution, and size properties to segment inflammatory areas and segmentation of edema. On the left is the original image; in the center is the original image with segmentation of severe inflammatory areas (shown in green); on the right is the original image and segmentation of edema (shown in green). The percent of the inflammatory area and edema area out of the total muscle area was calculated from these image analyses. Shown are representative samples including PF+Cripto after 7 days (A); empty PF after 23 days (B); and Wild-type control (C).

**Supplementary Figure S8. Histomorphology measurements of inflammation and edema in the tissue sections.** A quantitative analysis of the extent of edema and the severe inflammatory area was performed using computerized image analysis. No inflammation was observed in the WT group. In terms of Edema, the results in the non-WT groups were similar to the results in the WT group.
