# Supplementary material for: Development of a local controlled release system for therapeutic proteins in the treatment of skeletal muscle injuries and diseases
Source: Cell Death Dis. 2024 Jul 2;15(7):470. doi: 10.1038/s41419-024-06645-2 (PMC11219926; doi:10.1038/s41419-024-06645-2)
Supplement: Supplementary file 2 — Supplementary Figures [file 41419_2024_6645_MOESM2_ESM.pptx]

## Slide 1
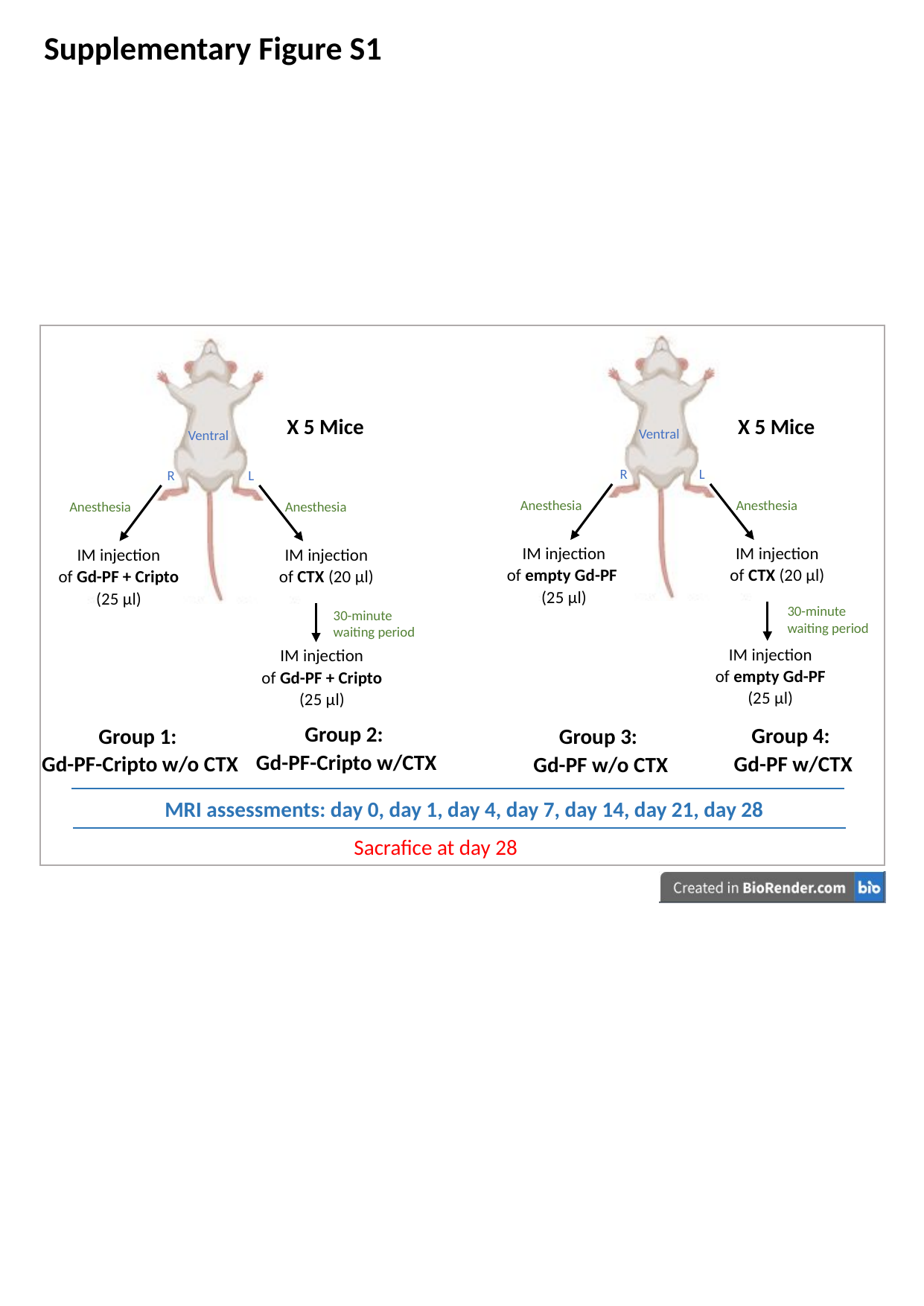

Supplementary Figure S1
X 5 Mice
X 5 Mice
Ventral
Ventral
L
R
L
R
Anesthesia
Anesthesia
Anesthesia
Anesthesia
IM injection
of CTX (20 μl)
IM injection
of empty Gd-PF
(25 μl)
IM injection
of CTX (20 μl)
IM injection
of Gd-PF + Cripto
(25 μl)
30-minute
waiting period
30-minute
waiting period
IM injection
of empty Gd-PF
(25 μl)
IM injection
of Gd-PF + Cripto
(25 μl)
Group 2:
Gd-PF-Cripto w/CTX
Group 4:
Gd-PF w/CTX
Group 1:
Gd-PF-Cripto w/o CTX
Group 3:
Gd-PF w/o CTX
MRI assessments: day 0, day 1, day 4, day 7, day 14, day 21, day 28
Sacrafice at day 28

## Slide 2
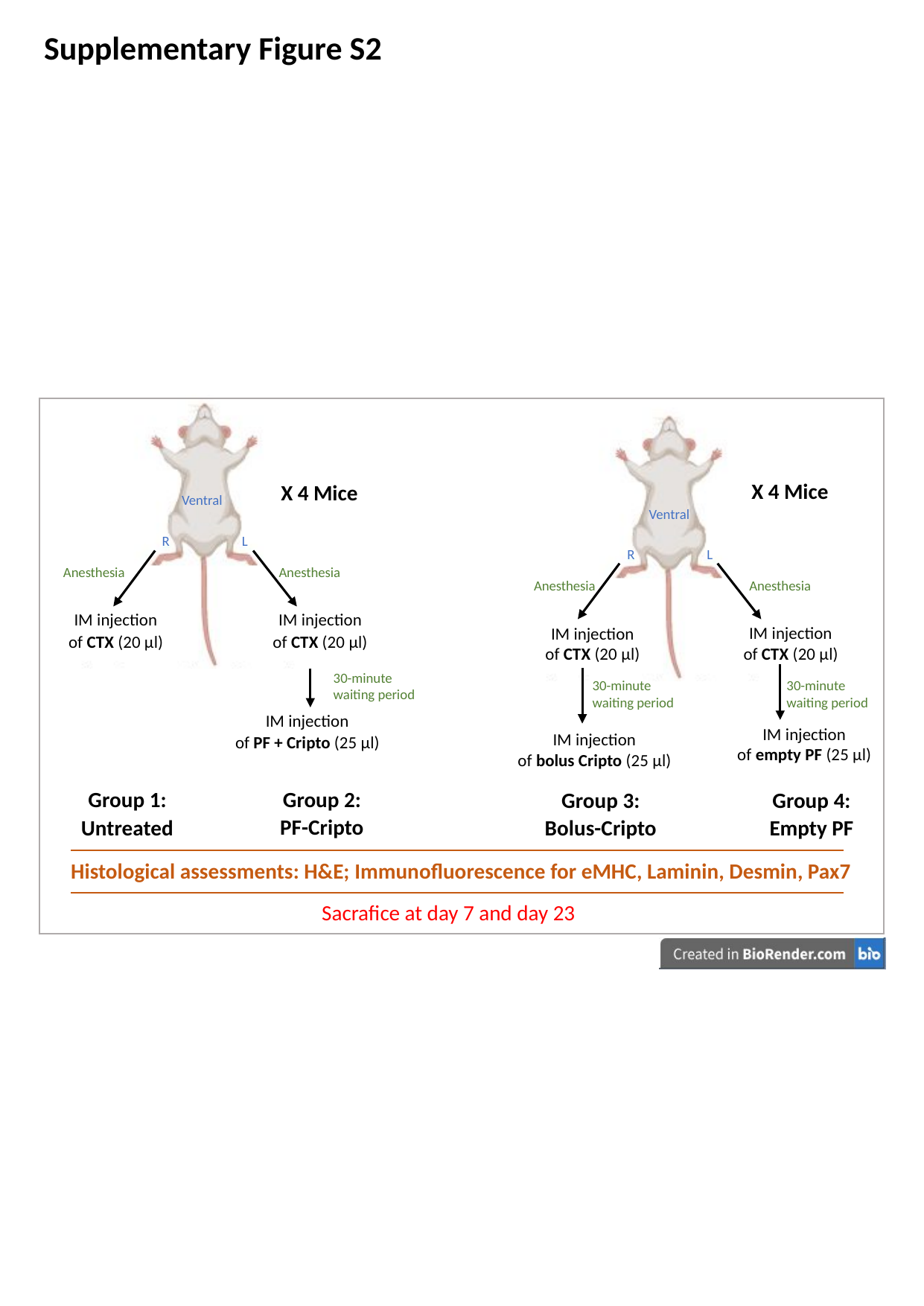

Supplementary Figure S2
X 4 Mice
X 4 Mice
Ventral
Ventral
L
R
R
L
Anesthesia
Anesthesia
Anesthesia
Anesthesia
IM injection
of CTX (20 μl)
IM injection
of CTX (20 μl)
IM injection
of CTX (20 μl)
IM injection
of CTX (20 μl)
30-minute
waiting period
30-minute
waiting period
30-minute
waiting period
IM injection
of PF + Cripto (25 μl)
IM injection
of empty PF (25 μl)
IM injection
of bolus Cripto (25 μl)
Group 2: PF-Cripto
Group 1: Untreated
Group 4: Empty PF
Group 3: Bolus-Cripto
Histological assessments: H&E; Immunofluorescence for eMHC, Laminin, Desmin, Pax7
Sacrafice at day 7 and day 23

## Slide 3
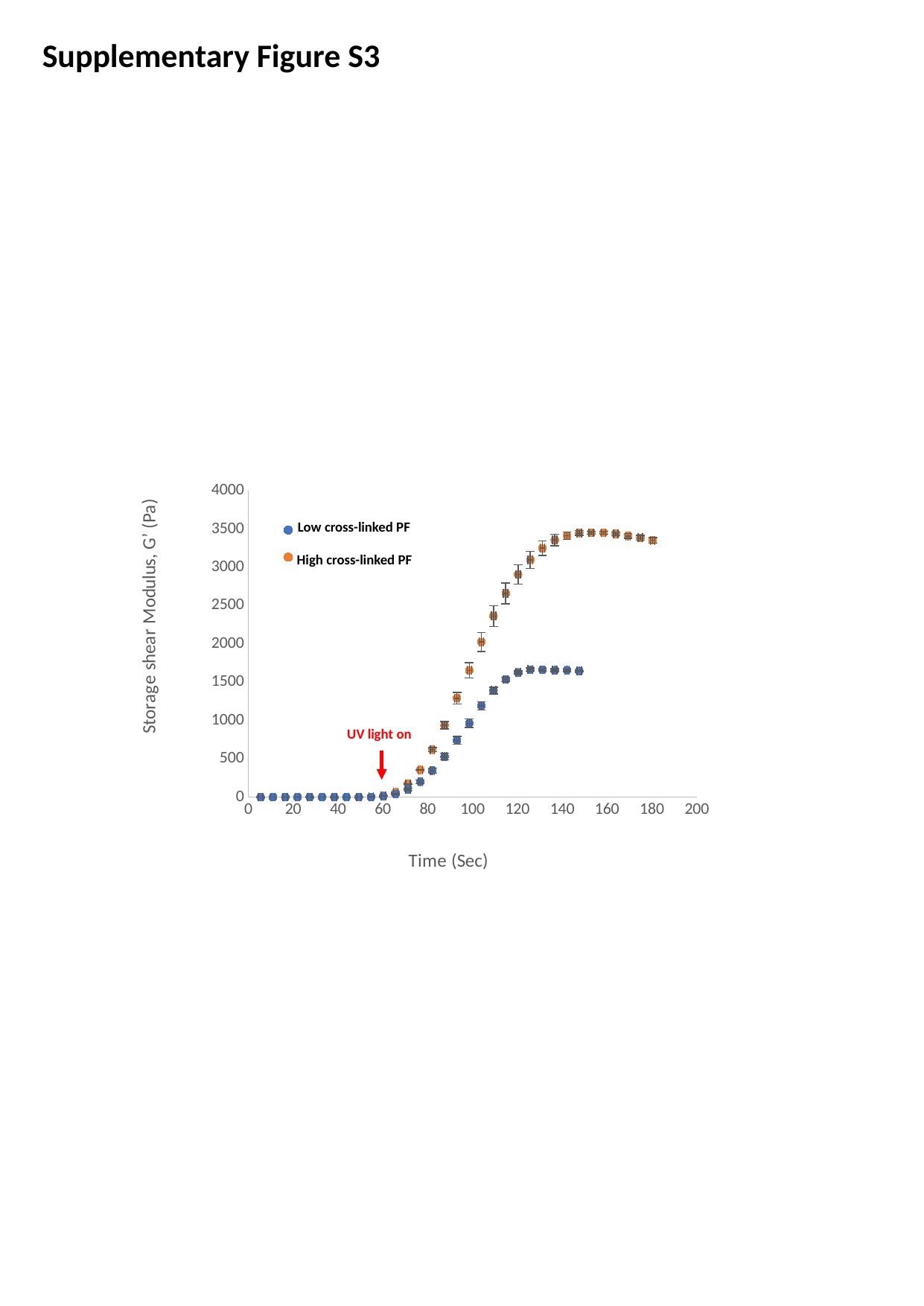

Supplementary Figure S3
### Chart
| Category | | |
|---|---|---|Low cross-linked PF
High cross-linked PF
UV light on

## Slide 4
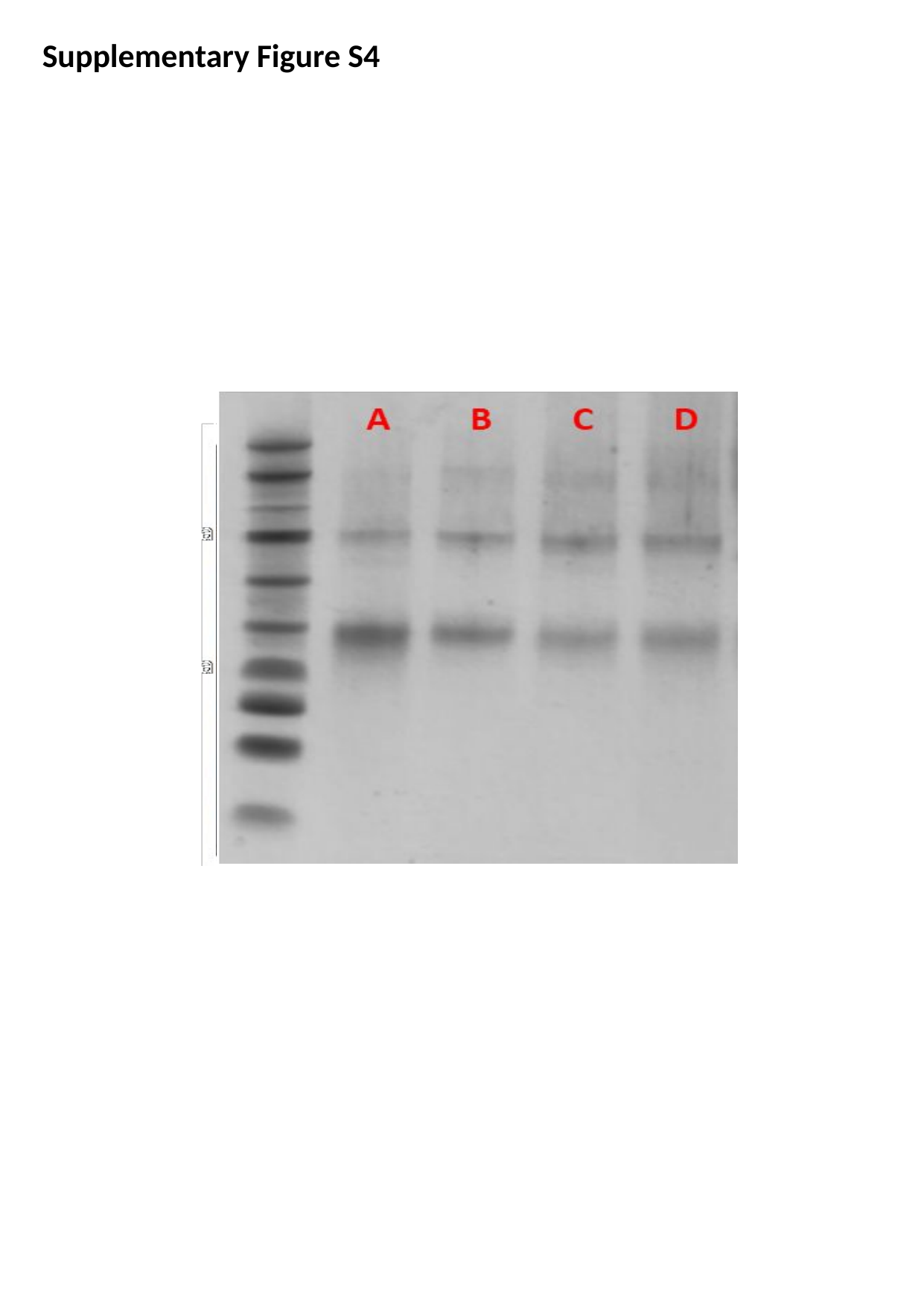

Supplementary Figure S4

## Slide 5
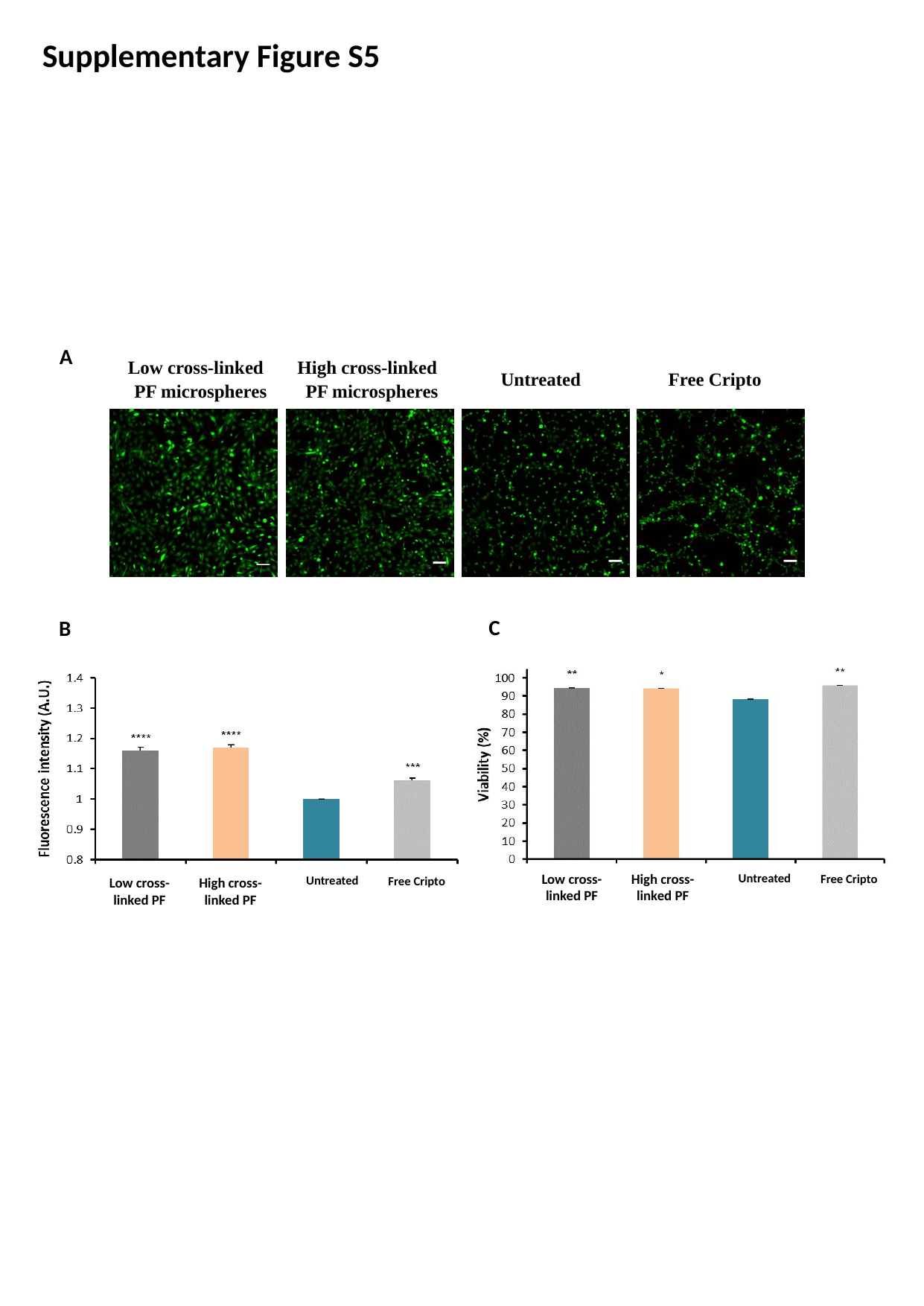

Supplementary Figure S5
A
| Low cross-linked PF microspheres | High cross-linked PF microspheres | Untreated | Free Cripto |
| --- | --- | --- | --- |
| | | | |
C
B
Untreated
PF microspheres G’=1600Pa
PF microspheres G’=3500Pa
Free Cripto
Low cross-
linked PF
High cross-
linked PF
Untreated
PF microspheres G’=1600Pa
PF microspheres G’=3500Pa
Free Cripto
Low cross-
linked PF
High cross-
linked PF

## Slide 6
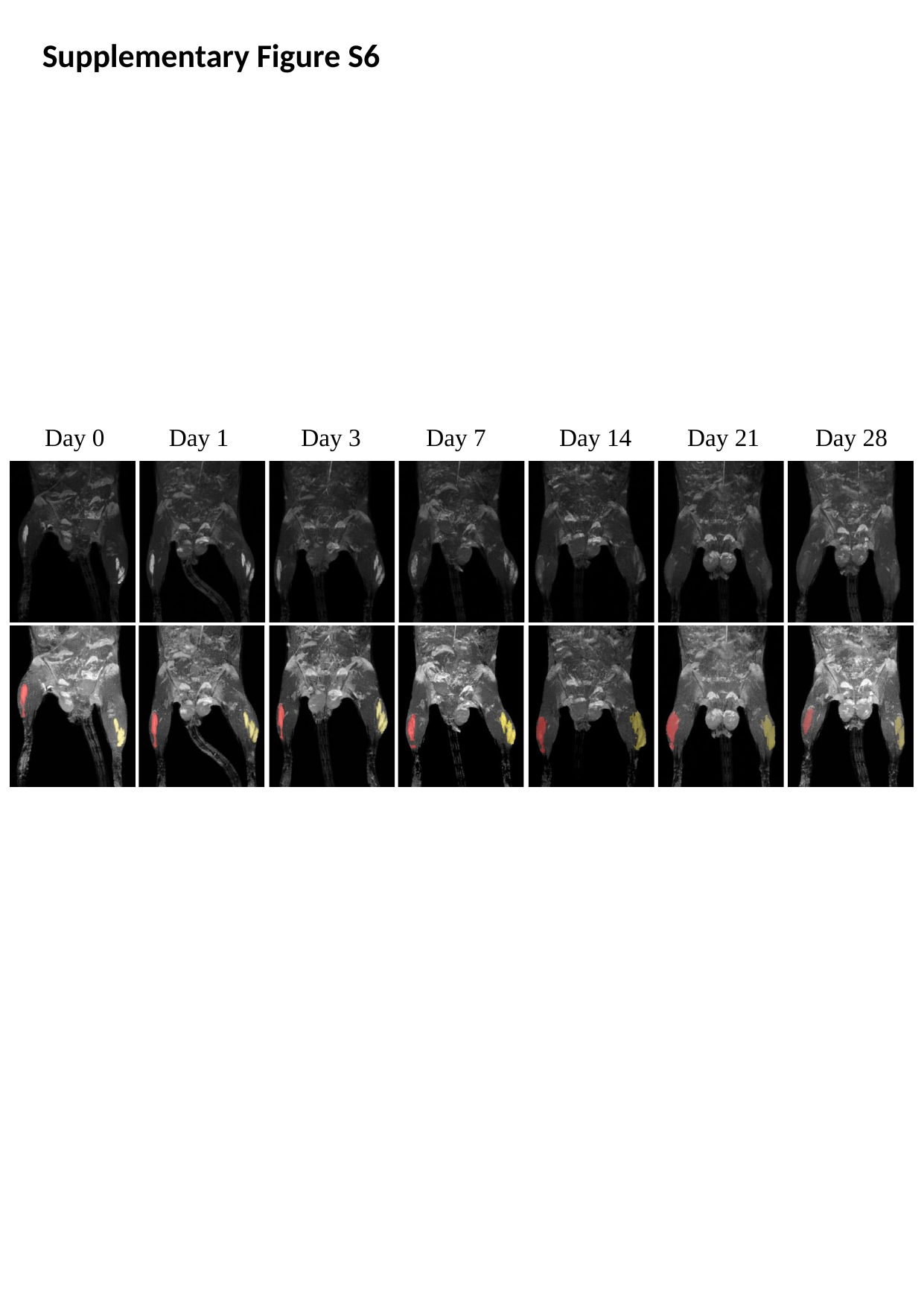

Supplementary Figure S6
Day 0
Day 1
Day 3
Day 7
Day 14
Day 21
Day 28

## Slide 7
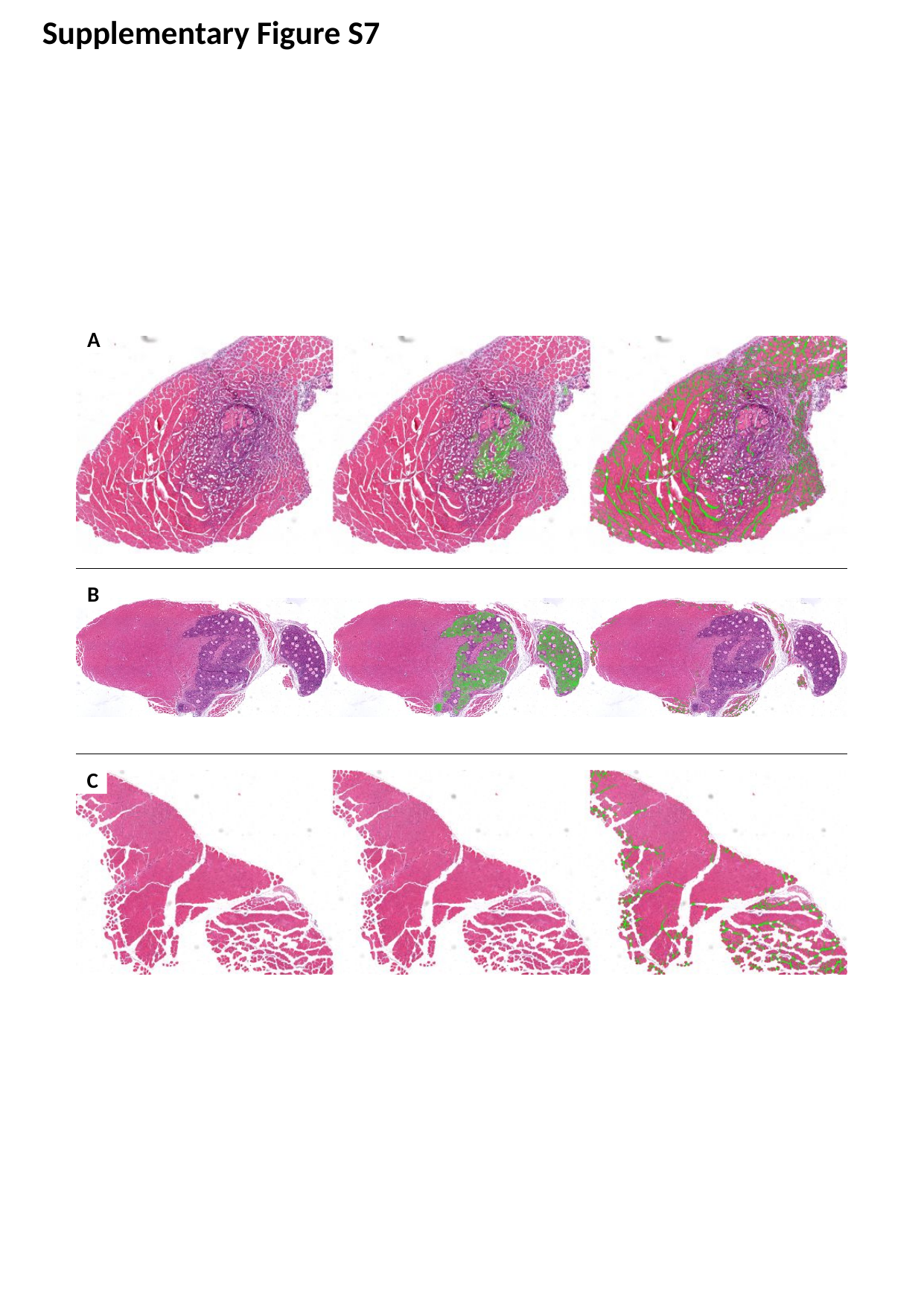

Supplementary Figure S7
A
B
C

## Slide 8
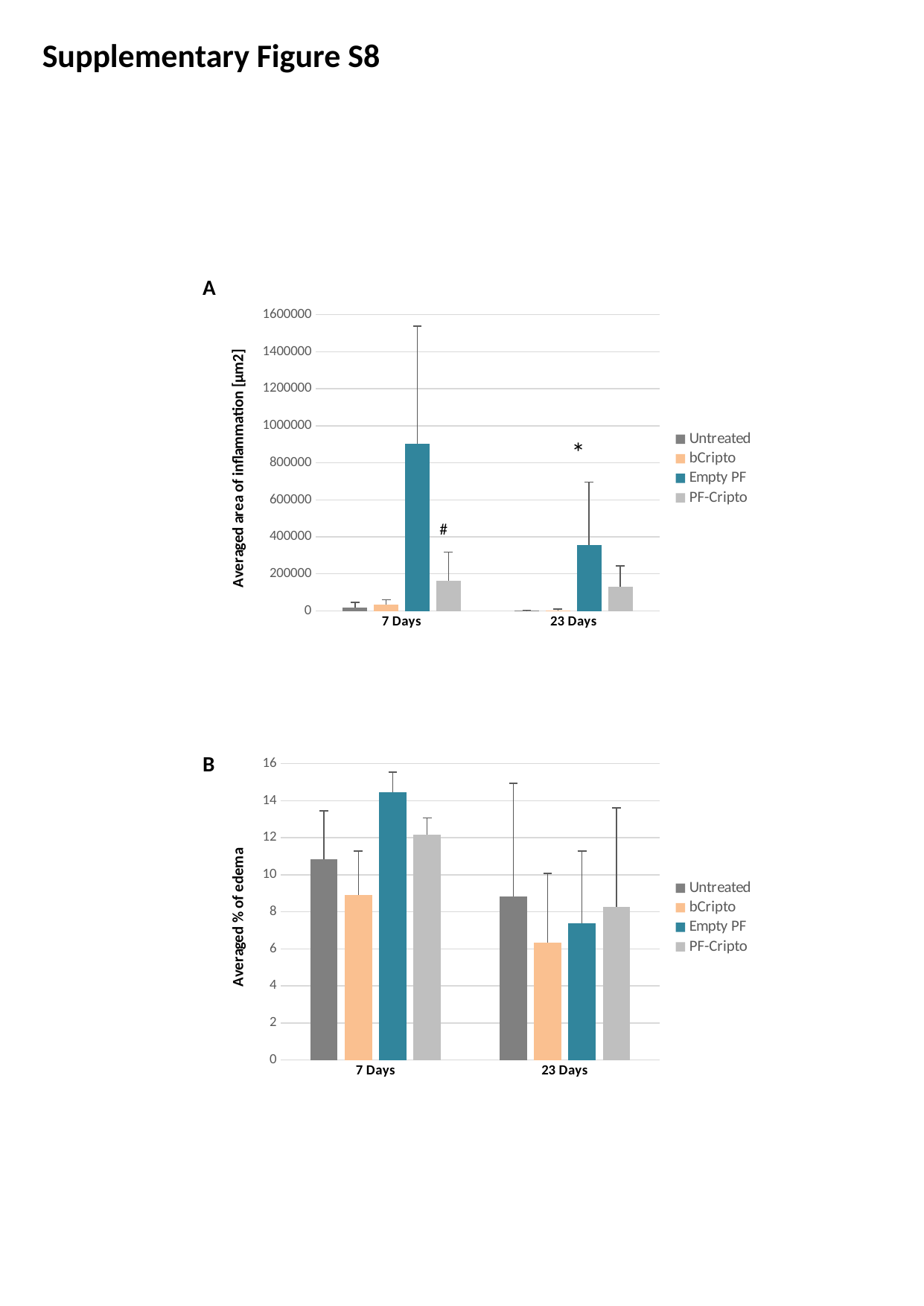

Supplementary Figure S8
A
### Chart
| Category | | | | |
|---|---|---|---|---|
| 7 Days | 17362.77 | 33408.51 | 903852.8 | 164354.58 |
| 23 Days | 1549.88 | 3361.36 | 355432.79 | 132596.87666666668 |*
#
B
### Chart
| Category | | | | |
|---|---|---|---|---|
| 7 Days | 10.85 | 8.91 | 14.46 | 12.17 |
| 23 Days | 8.84 | 6.34 | 7.38 | 8.26 |
